# Safety-constrained and runtime-assured LLM multi-agent decision making for intelligent vehicle systems

**DOI:** 10.1371/journal.pone.0357928

**Published:** 2026-10-30

**Authors:** Wei Dang, Lijun Gao

**Affiliations:** Beijing Information Technology College, Beijing, China; National University of Singapore, SINGAPORE

## Abstract

Large language models offer flexible semantic reasoning for intelligent-vehicle interfaces, but probabilistic outputs, multi-agent disagreement, and provider failures complicate safety-sensitive use. We developed SCI-Proposed-v1.0, a lightweight multi-agent semantic decision architecture that combines transparent risk-informed routing with an independent Safety Validator and deterministic local Runtime Fallback. The system produces canonical semantic actions rather than trajectories or direct vehicle-control commands. In a frozen confirmatory benchmark of 150 unseen Base Scenarios, each represented by three reviewed language variants, SCI-Proposed completed 360/450 inputs (80.0%), compared with 169/450 (37.6%) for EI-MAS and 155/450 (34.4%) for a Single LLM. Language variants were treated as repeated observations within each Base Scenario, and all methods used the same frozen model configuration. Base-scenario paired differences were 0.4244 (95% confidence interval, 0.3489–0.5000; p < 0.001) and 0.4556 (95% confidence interval, 0.3800–0.5311; p < 0.001), respectively. Complete Safety Requirement Satisfaction was 316/360 (87.8%) versus 101/360 (28.1%) for EI-MAS. In frozen ablation, removing the Safety Validator reduced its prespecified endpoint by 0.2767 (p < 0.001), and removing Runtime Fallback reduced represented-failure recovery by 0.3667 (p = 0.00195). Exact three-of-three canonical action-set stability was 27/30 (90.0%). In prespecified isolated-sandbox hardware-in-the-loop validation, Scientific Task Success was 25/30, Safety Enforcement Success was 7/7 applicable cases, and Runtime Recovery was 4/6 applicable cases. Across these complementary, prespecified stages, separating generative reasoning from independent safety validation and class-bounded local runtime recovery materially improved semantic-decision dependability under the tested qwen-plus/DashScope configuration, frozen semantic policy, and represented fault classes. These findings do not establish broader vehicle-system safety, physical-control reliability, arbitrary-fault recovery, or deployment readiness.

## 1. Introduction

Large language models (LLMs) can map natural-language instructions and structured context to useful task representations without task-specific parameter updates, and instruction tuning has improved their ability to follow user intent [1,2]. Agent frameworks extend this capability by interleaving reasoning and action, assigning roles, and coordinating multiple model calls [3]. These properties are attractive for intelligent-vehicle interfaces, where a request may need to be interpreted together with driver fatigue, vehicle energy state, diagnostic evidence, and the availability of runtime services.

The shift from a single call to an agent or multi-agent workflow changes both the opportunity and the engineering problem. Memory, reflection, role assignment, and structured inter-agent exchange can support tasks that are difficult to solve in one prompt [4–6]. In intelligent-vehicle research, Agent-Driver combined an LLM with tools, memory, reasoning, and reflection, while LMDrive connected language instructions with multimodal inputs in closed-loop driving [7,8]. Knowledge-driven and multimodal approaches have subsequently explored common-sense decision making, graph-structured reasoning, reasoning–decision alignment, conversational driving assistance, driving question answering, vision-language planning, and asynchronous planner assistance [9–15]. Multi-agent vehicle systems have also used shared memory, multi-step planning, and hierarchical cooperation [16,17]. Together, these studies show that language models can contribute at several vehicle-system abstraction levels.

Decomposition does not itself provide assurance. It introduces routing decisions, additional provider calls, intermediate schemas, arbitration rules, and termination conditions. A specialist can be unavailable, recommendations can conflict, and a response can be malformed or delayed. Even when the ordinary generative path completes, a linguistically plausible action set may omit a mandatory warning, contain an unsupported operation, or fail to request confirmation under uncertainty. SanDRA illustrates a complementary direction by applying reachability analysis outside the LLM to screen candidate automated-driving actions [18]. Its control-oriented object differs from the semantic actions studied here, but it exemplifies the broader principle that acceptance need not be decided by the model that generated the proposal.

The distinction between successful generation and admissible action is important because general AI-safety work identifies specification errors, side effects, unsafe exploration, and distributional shift as separate failure mechanisms [19]. Evaluations of tool-using agents likewise report long-tail risks, deficient risk awareness, prompt-injection vulnerability, difficulty following complex agentic constraints, and occasional harmful computer-use actions [20–24]. These studies do not evaluate vehicle functions, but they support a systems-level concern directly relevant to vehicle-associated agents: adding tools, roles, and external state expands both capability and the surface on which instructions, intermediate data, or runtime services can fail.

Two dependability failures should therefore be separated. First, an available candidate action set may violate an explicit semantic policy. This is a decision-validity problem even when every provider call succeeds. Second, the ordinary path may fail to return a usable candidate because of timeout, unavailable agents, malformed output, API failure, or represented conflict. This is a runtime-continuity problem. Independent validation is suited to the first failure; bounded local recovery may address selected instances of the second. Treating both as prompt-quality problems obscures their different triggers, mechanisms, and endpoints.

Runtime-assurance research provides an architectural precedent for this separation. The original Simplex architecture placed a complex advanced controller behind independent switching logic and a safer baseline controller [25]. Subsequent work has formalized safety filtering, robust and distributed extensions, neural-controller assurance, black-box controller handling, shielding, verified runtime monitoring, and adaptive Simplex variants [26–36]. These approaches differ in assumptions and proof obligations, but share a useful design principle: performance generation and assurance are distinct functions, and any fallback claim must be bounded by its monitored conditions and response model.

The present study adapts that principle at a semantic action boundary rather than a motion-control boundary. A semantic cockpit layer can express warnings, service recommendations, confirmations, navigation intent, and denial of prohibited requests without producing waypoints, steering, braking, throttle, or CAN commands. This middle layer permits a generated action set to be canonicalized and checked against a frozen policy before any engineering transport. It also permits failures of the provider-dependent path to be handled from local structured facts without consulting evaluator-only answers. The resulting claim is necessarily narrower than verified autonomous control: semantic policy satisfaction does not prove trajectory safety, physical execution, or compliance with a functional-safety lifecycle.

The resulting gap is not whether LLMs can participate in vehicle-related reasoning, nor whether safety filters or runtime monitors are useful in isolation. It is whether independent semantic Safety Validation and deterministic, class-bounded Runtime Fallback make separately measurable contributions within the same frozen LLM multi-agent architecture. Addressing that question requires more than an overall accuracy comparison: the method, policy, ontology, Ground Truth, failure classes, and statistical endpoints must be frozen; the assurance components must be ablated independently; repeated executions must be evaluated on canonical semantics; and engineering integration must remain downstream of scientific scoring.

We investigated whether this layered architecture improves semantic Task Success and Safety Requirement Satisfaction relative to a Single LLM and a multi-agent baseline without the independent safety/fallback layer. We further tested which components contributed under frozen ablations, whether canonical action sets remained stable across repeated executions, and whether the scientific decision boundary was preserved during isolated-sandbox HIL integration. The study used a development Pilot excluded from confirmatory inference, a frozen 150-scenario Formal dataset with human-reviewed Ground Truth, Base-scenario clustered analyses prespecified and frozen before outcome inspection, and a prespecified 30-case HIL validation. This staged design examined system effectiveness, component attribution, repeatability, and integration containment without treating them as interchangeable outcomes. The results support the Safety Validator and Runtime Fallback within the represented conditions, while placing strict limits on claims about unsafe-action reduction, general fault recovery, gateway delivery, and physical actuation.

## 2. Related Work

### 2.1 LLM-based decision making for intelligent vehicles

Vehicle-focused language-model research now spans perception-linked question answering, high-level reasoning, behavior planning, cooperative decision making, and closed-loop control. Agent-Driver organized tool use, memory, reasoning, planning, and reflection around an autonomous-driving agent [7]. LMDrive coupled language instructions to sensor observations in a closed-loop end-to-end system [8]. DiLu framed driving as knowledge-driven reasoning with memory and reflection, emphasizing generalization beyond purely data-driven policies [9]. These systems established that language models can structure driving knowledge and decisions, but they also demonstrate that the term “action” can denote very different outputs—from an explanation or high-level maneuver to a trajectory-related result. Rapid development is also occurring in adjacent physical-decision layers: integrated reinforcement-learning frameworks combine behavioral decision making, path planning, and motion control for high-speed cruising, while interaction-aware trajectory prediction explicitly models vehicle interactions and predictive uncertainty for downstream motion planning [37,38]. These studies underscore both the pace of intelligent-vehicle research and the importance of keeping semantic-decision assurance distinct from trajectory and control assurance.

Multimodal work broadens that range. DriveLM introduced graph visual question answering across perception, prediction, and planning [10]. RDA-Driver explicitly addressed misalignment between generated reasoning and downstream decisions [11]. Dolphins used video or images, instructions, and historical control signals for several driving tasks [12]. LingoQA provided a large, human-annotated driving question-answering benchmark and a learned truthfulness evaluator [13]. VLP used vision-language representations to support planning [14], while AsyncDriver decoupled slower LLM inference from a faster real-time planner [15]. Recent work has also separated open-ended instruction interpretation from lower-level execution: Liu et al. used an LLM to generate scripts that scheduled model-predictive-control-based planners from real-time feedback, making the semantic-to-control interface explicit rather than collapsing the two layers [39]. These studies are relevant because they expose recurring interfaces among language, structured scene representations, planning, and execution. They do not, however, establish that a semantically plausible response satisfies an independently defined operational policy.

Multi-agent vehicle studies emphasize cooperation rather than only multimodality. KoMA incorporated multiple agents, shared memory, multi-step planning, and ranking-based reflection [16]. CCMA coordinated individual, regional, and global levels for merging decisions [17]. Such organization can improve contextual coverage or cooperation, but a larger number of model-mediated components also creates more failure points. The scientific question is therefore not simply whether multi-agent decomposition can improve a mean outcome. It is whether contributions from coordination can be distinguished from contributions made by mechanisms that independently constrain or recover the output.

The present work does not reproduce or directly benchmark these autonomous-driving systems. It addresses canonical semantic cockpit and safety actions: issuing a warning, recommending a break, navigating to a destination class, requiring confirmation, denying a diagnostic-code-clear request, or invoking a local fallback. It does not perceive road geometry, generate waypoints, estimate collision-free trajectories, or command vehicle motion. This narrower boundary permits controlled evaluation of semantic correctness and policy compliance before any downstream navigation or control subsystem. Comparisons in this study therefore concern frozen semantic decision architectures using the same runtime inputs and action representation, not performance against published driving stacks.

### 2.2 LLM multi-agent architectures

General LLM-agent work provides mechanisms relevant to semantic vehicle interfaces. ReAct interleaved reasoning with task actions [3], Generative Agents combined memory, reflection, and planning [4], CAMEL formalized role-playing communication between agents [5], and AutoGen provided configurable multi-agent conversations, tools, and human participation [6]. These frameworks show how role specialization and explicit message exchange can decompose complex tasks. They also expose coordination as a distinct source of system behavior: routing can omit a relevant specialist, agents can disagree, intermediate schemas can fail, and termination logic can produce a result even when required information is absent.

The vehicle-focused systems above instantiate several of these ideas, including tool invocation, shared memory, reflection, and inter-agent planning [7,9,16,17]. Yet the architectural mechanism and the assurance claim must remain separate. A dispatcher can make role selection inspectable without proving that the chosen roles are sufficient. Consensus can reduce disagreement without proving that the agreed action is permitted. Reflection can revise a candidate without guaranteeing termination or provider availability. A structured schema can make parsing easier without preventing omission of a mandatory action.

Multi-agent organization should therefore not be equated with assurance. More agents may increase contextual coverage, but additional calls and interfaces enlarge the failure surface. A credible evaluation must separate benefits due to specialization and routing from benefits due to components that constrain or replace generative outputs. SCI-Proposed uses a fixed dispatcher plus fatigue, diagnosis, and scene roles, with a frozen schema and arbitration order. The study does not claim a novel general conversation protocol. Instead, it tests risk-informed coordination as supporting architecture and separately ablates independent, non-generative assurance components.

### 2.3 Safety verification for LLM-generated vehicle decisions

Concrete AI-safety problems include wrong objectives, side effects, unsafe exploration, and behavior outside the training distribution [19]. Tool-using agents add operational risks because model outputs can select external functions and alter state. ToolEmu used an LLM-emulated sandbox and automated safety evaluator to identify high-stakes, long-tail failures without implementing every real tool environment [20]. R-Judge evaluated whether agents recognized safety risks across interactive traces [21]. AgentDojo formalized utility and security tests for agents operating on untrusted tool-returned data [22]. These studies motivate evaluation at the action and environment boundary rather than reliance on response fluency alone.

More recent benchmarks extend the concern from adversarial prompts to instruction complexity and harmful computer use. AgentIF evaluates long agentic instructions containing many interacting constraints and tool specifications [23]. OS-Harm covers deliberate misuse, prompt injection, and model misbehavior in computer-use tasks [24]. Their environments are not vehicles, and their outcome definitions cannot be transferred directly to this study. Their relevance is narrower: an agent can complete parts of a task while violating a constraint, and capability evaluation can obscure a safety failure unless utility and admissibility are scored separately.

In vehicle settings, SanDRA placed formalized traffic rules and reachability analysis outside the generative model to remove unsafe candidate actions [18]. That work evaluates driving actions with a vehicle-dynamics model; the present validator instead evaluates canonical semantic actions against a frozen ontology and policy. Its relevant question is whether a candidate contains every minimum required warning, confirmation, or service response and excludes explicitly forbidden operations. A deterministic semantic validator can canonicalize aliases, reject malformed or unknown actions, resolve mutually exclusive intents, and add context-required interventions without claiming that a downstream trajectory is safe. This distinction separates policy satisfaction from model fluency and from physical vehicle safety. It also permits an all-required-actions scenario endpoint to coexist with descriptive action-level recall and precision.

### 2.4 Runtime assurance and failure recovery

Simplex-style runtime assurance decouples a high-performance component from a simpler mechanism responsible for keeping execution within an acceptable region. The original architecture supported safe online controller upgrades through independent decision logic and a baseline controller [25]. Contemporary overviews describe a broader family of safety filters and runtime-assurance patterns for complex controllers [26]. Robust Simplex addresses both cyber and physical failures [27], while distributed Simplex extends coordination to multi-agent systems [28]. These variants clarify that a runtime-assurance claim depends on explicit assumptions about the monitored plant, the baseline behavior, and switching logic.

Learning-enabled systems have prompted further variants. Neural Simplex supplies runtime assurance around neural controllers and permits return to an adapted advanced controller [29]. Black-Box Simplex reduces reliance on internal knowledge of the advanced and baseline controllers by validating candidate command sequences at runtime [30]. Shielding synthesizes a reactive mechanism that blocks actions violating a temporal specification [31], and formal-methods approaches combine verified runtime monitoring with learning [32]. SOTER integrates a runtime-assurance mechanism into a robotics programming framework [33]. Runtime monitoring has also been evaluated for an end-to-end learning-based model vehicle [34] and formalized for partially observed Markov decision processes [35]. Adaptive Simplex work continues to explore verified switching and recoverable-region extension [36].

These approaches are not interchangeable with the present semantic validator. Most reason about control signals, dynamics, reachability, or formal state models. SCI-Proposed does not transfer their control-theoretic guarantees to LLM outputs. It adopts only the architectural separation: the generative path proposes, an independent mechanism checks, and a bounded fallback may respond when the ordinary path fails. Runtime recovery is also distinct from validation of a completed decision. A validator acts on an available candidate set; a fallback acts when the ordinary path raises a represented exception or cannot return a usable candidate. Recovery from timeout or malformed output does not imply recovery from semantic conflict, contradictory state, arbitrary corruption, or every unavailable-agent target. The study therefore treats Runtime Fallback as conditional and class-bounded, evaluates it through a frozen failure ontology, and avoids a universal fault-tolerance claim.

### 2.5 Positioning of the present study

Existing studies have investigated LLM driving agents, multimodal planning, multi-agent cooperation, external verification of generated driving actions, agent-safety evaluation, and runtime-assurance architectures. These lines operate at different abstraction levels and use different evidence. The present study places their relevant design ideas around a semantic action boundary. The ordinary LLM path proposes a canonical action set; an independent scientific Safety Validator enforces the frozen semantic policy; deterministic local Runtime Fallback handles only represented runtime failures; and a later HIL interlock contains engineering transport after scientific scoring. The scientific validator and engineering interlock are complementary but not interchangeable.

This positioning leads to four separate empirical questions. First, does the complete architecture improve semantic Task Success and complete Safety Requirement Satisfaction against frozen comparators? Second, do Safety Validation, Runtime Fallback, and Risk Assessment make independently measurable contributions under contemporaneous ablation? Third, are final canonical action sets repeatable across new executions when raw language is ignored? Fourth, does an isolated-sandbox HIL path preserve rather than rewrite the scientific decision boundary? Answering these questions requires complementary Formal Main, ablation, Decision Stability, and HIL evidence rather than pooling them into one success rate. The contribution is thus an evaluated separation of generative reasoning, semantic assurance, bounded recovery, and engineering containment—not a claim of autonomous-driving control.

## 3. Materials and Methods

### 3.1 Study scope and architecture

The study evaluated semantic decisions for intelligent-vehicle interfaces. Each runtime input contained a natural-language instruction plus structured driver, vehicle, diagnostic, context, runtime-status, and fault-injection fields. The scientific output was a canonical set of semantic actions. Neither the model nor the scientific evaluator generated CAN identifiers, UDS commands, trajectories, steering, braking, throttle, or other low-level control. A mock semantic-action executor was used in the Formal software experiments.

The SCI-Proposed-v1.0 path comprised: (1) a Context Builder; (2) transparent Risk Assessment; (3) risk-informed routing to fatigue, diagnosis, and scene specialists; (4) multi-agent arbitration; (5) an independent Safety Validator; and (6) conditional Runtime Fallback for represented runtime failures. Frozen Ground Truth entered only the evaluator after the final canonical action set had been produced. Scientific scoring was completed before the HIL engineering path, which added a separate interlock, containment guard, Adapter v1.1, semantic mapping check, and isolated vendor sandbox (Fig 1).

**Fig 1:**
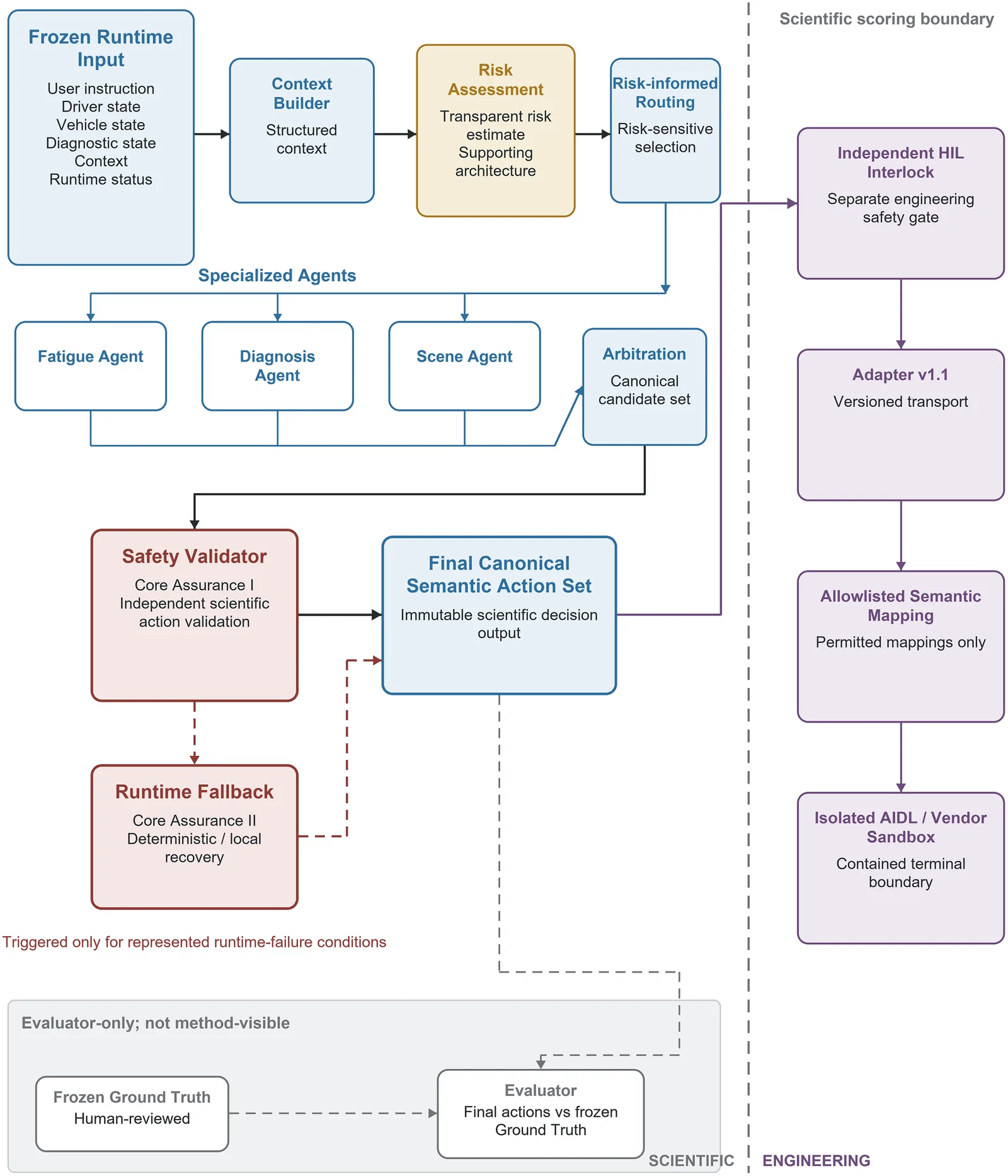
Architecture of the safety-constrained and runtime-assured semantic decision framework. Frozen runtime inputs are structured by the Context Builder, assessed by the transparent Risk Assessment module, routed to specialized agents, arbitrated, and independently validated before producing the final canonical semantic action set. Risk-informed routing is supporting architecture. The scientific Safety Validator is Core Assurance I; Runtime Fallback is Core Assurance II and is triggered only for represented runtime-failure conditions rather than acting as a mandatory serial stage. Frozen Ground Truth enters only the evaluator branch and is not method-visible. Scientific scoring is completed before the engineering boundary. The independent HIL interlock is distinct from the scientific Safety Validator, and only versioned allowlisted mappings may proceed through Adapter v1.1 to the isolated AIDL/vendor sandbox.

The method tree, Action Ontology v1.0, action aliases, Safety Policy v1.0, Formal configuration, statistical analysis plan, and scientific source-tree identity were frozen and independently verified before Formal outcome inspection.

### 3.2 Context construction and semantic action ontology

The Context Builder deep-copied method-visible runtime fields and rejected any input containing evaluator-oracle data. The Action Ontology defined ordinary navigation classes, urgent safe-stop navigation, service and charger navigation, warning and recommendation actions, confirmation and denial actions, and recovery actions. Recognized unsafe-intent tokens such as direct diagnostic-code clearing, warning disablement, unsafe override, raw CAN writing, and unsupported execution were included for evaluation and blocking but were never executable actions.

The ontology separated user intent from the safe system response. For example, a state-only report could have no requested operation while Ground Truth still required a warning or service recommendation. NAVIGATE_TO_REST_AREA was distinct from urgent NAVIGATE_TO_SAFE_STOP, and no action alias could cross that semantic boundary. Standard diagnostic trouble codes were separated from research-only synthetic diagnostic conditions.

The Action Ontology and Safety Policy were research-constructed from the prespecified task boundary, Pilot-stage error analysis, the frozen action schema and aliases, and explicit required/forbidden-action rules, and were frozen before Formal execution. The documented role of the domain experts was Ground Truth annotation and adjudication; no separate expert validation of the Action Ontology or Safety Policy is claimed. These artifacts are therefore versioned task specifications rather than a complete vehicle-function taxonomy. Extension to a new function, diagnostic condition, or semantic action would require prospective definition of the canonical token, aliases, and schema; method-visible preconditions; required, forbidden, and mutually exclusive combinations; conflict and precedence rules; fallback eligibility; domain-expert review of the proposed addition; validator, fallback, unit, and adversarial tests; a version increment and new freeze; and independent revalidation before use. Until that process is completed, unregistered semantics remain unknown and non-executable. The current study did not validate this extension workflow on previously unseen vehicle functions.

### 3.3 Transparent risk assessment and routing

Risk Assessment used deterministic rules over method-visible facts. Four ordered levels were used: Low, Medium, High, and Critical. Features included fatigue level, driving duration, confidence, diagnostic severity and consistency, abnormal state without a standard diagnostic code, battery state of charge, cabin load, requested unsafe intent, and the primary injected failure. Prespecified thresholds included low state of charge at 20%, critical state of charge at 8%, long driving at 120 minutes, very long driving at 180 minutes, and low driver-state confidence below 0.6.

Base routing assigned the dispatcher and added fatigue, diagnosis, or scene agents when the corresponding context was present. Risk-informed routing could add only context-justified specialists and recorded the exact reasons for each addition. Risk Assessment received neither Scenario ID nor Ground Truth. Because risk-informed routing was expected to support coordination rather than independently enforce safety, its causal contribution was evaluated separately in the ablation experiment.

These thresholds were prespecified operational anchors for the frozen research benchmark, not universal physiological, regulatory, clinical, or vehicle-control limits. They were not optimized against Formal outcomes, and this study did not perform a threshold-sensitivity analysis. Their methodological role was to provide transparent deterministic stratification within the controlled benchmark; the study did not seek to validate the numerical cut points themselves. Their interpretation and resulting risk classes may change with vehicle type, driver population, sensor calibration, jurisdiction, or operational design domain; transfer would therefore require prospective calibration, sensitivity analysis, and a new frozen validation before any deployment claim.

### 3.4 Agent invocation, arbitration, and comparators

Each invoked role received the same method-visible context encoded as sorted JSON and a system instruction to return JSON with actions and activated agents selected from the frozen schema. The instruction prohibited raw CAN/UDS output and attempts to disable the constraints. Specialist responses were collected and deduplicated. Arbitration applied a frozen safety-priority order to candidate actions and could add narrowly specified actions for transparent risk reasons, including a break recommendation after very long driving or a warning and confirmation for an abnormal state without a standard diagnostic code.

The Single LLM comparator made one model call and returned its structured semantic decision without the proposed risk, safety-validation, or local-fallback components. EI-MAS was an author-implemented, architecture-matched multi-agent comparator, not a reproduction of a published autonomous-driving framework. It shared with SCI-Proposed the underlying LLM/provider, sampling configuration, runtime-input representation, semantic Action Ontology/schema, and dispatcher/specialist-agent framework. EI-MAS used the base deterministic routing derived from runtime facts, combined specialist actions, and contained neither the independent SCI-Proposed Safety Validator nor Runtime Fallback; SCI-Proposed could additionally use its frozen Risk Assessment to add context-justified specialists or arbitration actions. The comparison therefore evaluated complete frozen architectures, not token-matched prompts, compute-matched systems, or an equal LLM-call budget. Both comparators were research-constructed controls for this semantic task; they are not exhaustive state-of-the-art production baselines and do not establish general superiority over published multi-agent or autonomous-driving systems. Their role was to isolate system-level differences within the same frozen input and action representation.

### 3.5 Independent Safety Validator

The Safety Validator canonicalized aliases, rejected unknown or malformed actions, removed recognized unsafe intents, resolved mutually exclusive window actions to confirmation, blocked direct diagnostic-code clearing, denied model-override attempts, and added context-required interventions. These included fatigue warnings, break recommendations, vehicle-fault warnings, service recommendations, low-energy constraints, and confirmation for uncertain abnormal states. If the validated set was empty, the validator returned NO_VEHICLE_ACTION. It never received Scenario ID, Ground Truth, or draft annotations.

Safety validation was defined at two levels. Complete Safety Requirement Satisfaction required every Ground-Truth safety action for an input and no unsafe action. Action-level micro recall and precision counted individual required actions. These measures were kept distinct because a response could achieve high action recall while failing the all-required-actions criterion, and additional non-forbidden actions could reduce precision without making the decision unsafe.

The Safety Validator and evaluator Ground Truth intentionally shared the frozen semantic specification at the level of action vocabulary, minimum required actions, forbidden actions, and safety-requirement rules because the endpoint measured compliance with that explicit policy. This specification-level alignment defines the benchmark target; it does not establish an independent external safety criterion. Procedural and informational independence were nevertheless maintained: domain experts independently annotated and adjudicated Ground Truth, preliminary AI labels and all method outputs were hidden, the oracle was frozen before Formal execution, and the Validator received no Scenario ID, Ground Truth field, draft annotation, or query path to the evaluator. Accordingly, this endpoint quantifies enforcement within the prespecified specification rather than external safety validity.

### 3.6 Deterministic local Runtime Fallback and injected failures

Runtime Fallback was activated only after represented exceptions when the component was enabled. It always recorded FALLBACK_LOCAL_POLICY and then derived bounded actions from local facts, such as fatigue warning, break recommendation, safe-stop or rest-area navigation, fault warning, service navigation, charger navigation, or limitation of nonessential actions. If no context-specific action applied, it returned NO_VEHICLE_ACTION.

Each Failure Base Scenario contained exactly one primary injected failure. The frozen Fault Injection Ontology distinguished MALFORMED_OUTPUT, LLM_TIMEOUT, AGENT_UNAVAILABLE, AGENT_CONFLICT, INVALID_EXECUTION_REQUEST, MODEL_OVERRIDE, API_FAILURE, CONTRADICTORY_STATE, and INVALID_SEMANTIC_ACTION. Other hazards in a scenario were treated as background facts. Fallback was expected for malformed output, timeout, unavailable agent, invalid execution request, API failure, and invalid semantic action; conflict, model override, and contradictory state had policy-specific handling and were not automatically labeled fallback-expected. Results were interpreted only for the represented classes.

Unknown, simultaneous, compound, and arbitrary failures were not experimentally validated. If an input or failure lies outside the frozen ontology, the present implementation can reject unknown or malformed actions, but this study does not establish complete detection or recovery for every compound runtime state. A practical extension should route unrecognized or conflicting states to a fail-closed disposition that withholds execution, preserves the trace, and escalates to a human or independently verified subsystem; that behavior is a design requirement for future validation, not a measured outcome of the current experiments.

### 3.7 Development Pilot and Formal dataset

A 30-scenario Development/Pilot Set was used for platform validation and pre-freeze refinement. Phase 0 completed 18/18 calls and Phase 1 completed 90/90 calls. Pilot observations motivated general clarification of ontology, explicit unsafe opportunities, and requested-action semantics. They were permanently excluded from confirmatory inference and had zero overlap with the Formal dataset.

The Formal dataset contained 150 unseen Base Scenarios: 30 each in Normal, Fatigue, Diagnosis, Compound, and Failure categories. Difficulty was balanced at 50 Easy, 50 Moderate, and 50 Hard bases. Each base had three reviewed language variants with identical structured facts, producing 450 Language Inputs. The three variants were repeated observations within the same Base Scenario, not independent scenarios. The complete sequence from Pilot through Formal Main, ablation, Decision Stability, HIL preparation, and Formal HIL is shown in Fig 2.

**Fig 2:**
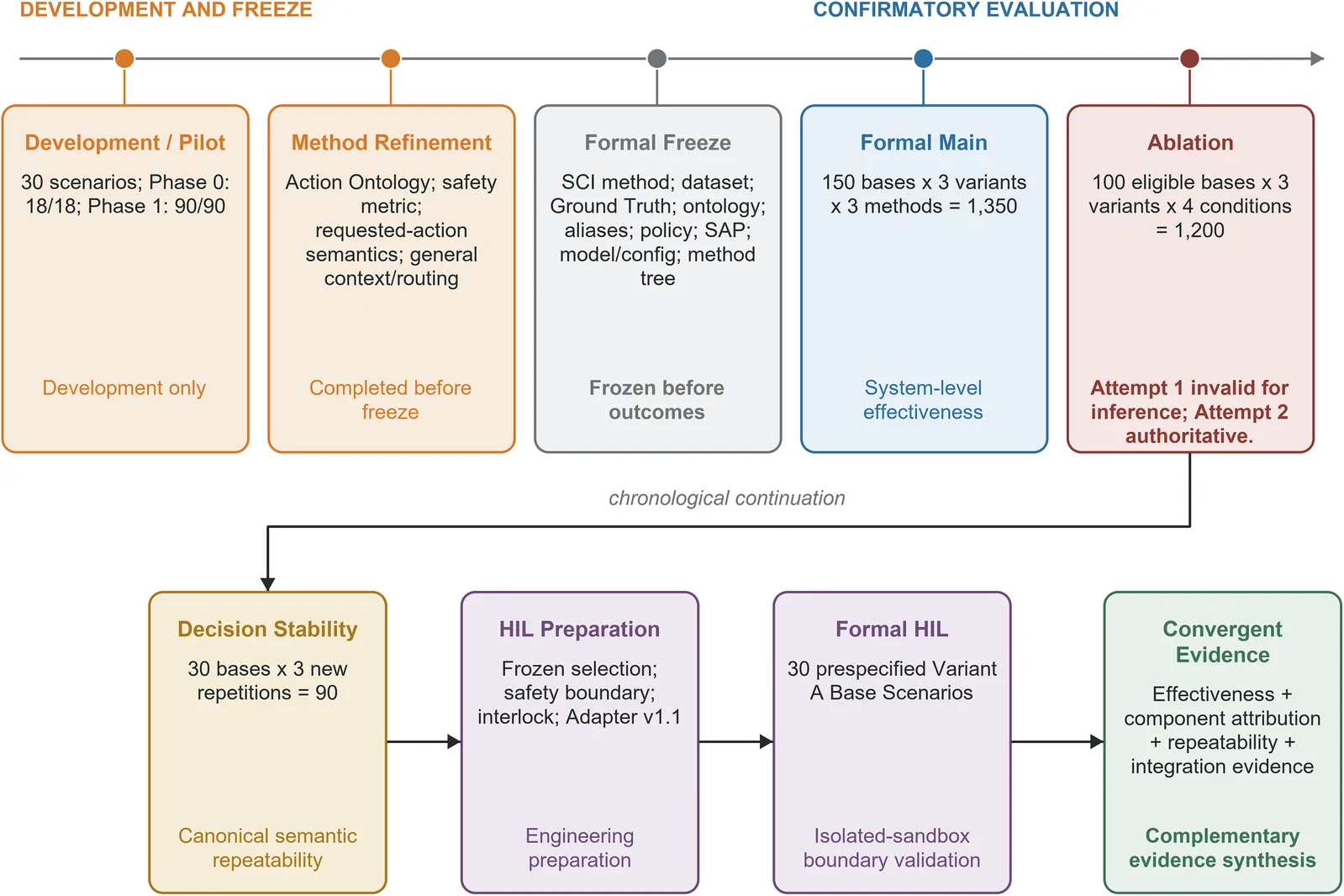
Frozen experimental design and complementary evaluation stages. The 30-scenario Development/Pilot Set completed Phase 0 (18/18) and Phase 1 (90/90) and informed only general pre-freeze refinement; Pilot observations were excluded from confirmatory inference. The resulting method, ontology, policy, Ground Truth, dataset, model/configuration, and statistical plan were frozen before Formal outcomes. Formal Main comprised 150 Base Scenarios × 3 language variants × 3 methods (1,350 runs). Ablation comprised 100 eligible bases × 3 variants × 4 conditions (1,200 Attempt-2 runs); Attempt 1 is retained but invalid for inference, whereas Attempt 2 is authoritative. Decision Stability comprised 30 bases × 3 new repetitions (90 runs), followed by frozen HIL preparation and 30 prespecified Variant A Formal HIL cases. These stages provide complementary evidence for system-level effectiveness, component attribution, canonical semantic repeatability, and isolated-sandbox integration.

### 3.8 Ground Truth construction, independent review, and consensus adjudication

Scenario construction and AI-assisted preliminary annotation were completed before expert review. Reviewer A assessed the scenarios from an intelligent-vehicle/automotive-systems perspective, whereas Reviewer B assessed them from an AI/software-engineering perspective. Preliminary AI annotation values were not shown to either reviewer during independent first-pass annotation. Each reviewer received the same Base Scenario identifiers and facts where applicable, category, difficulty, Variants A/B/C, explicit requested action, driver state, vehicle state, diagnostic state, full context, runtime status, and Primary Fault Injection. The corresponding annotation fields were empty and covered scenario validity, clarification need, risk level, required and forbidden actions, safety-intervention requirement, Failure-only fallback expectation, requested-action validity, paraphrase equivalence, confidence, and comments. Neither reviewer had access to AI draft annotation values, Safety Validator output, Pilot performance, Formal results, or outputs from Single LLM, EI-MAS, or SCI-Proposed.

Reviewer A and Reviewer B completed their first-pass annotations independently before cross-review. Field-level disagreements were then identified in 64 of the 150 Base Scenarios (42.7%). The two reviewers jointly resolved these cases through consensus discussion using the frozen risk anchors, minimum-required-action rules, required/forbidden-action rules, fallback criteria, paraphrase-equivalence criteria, and other prespecified annotation criteria. No method result was available during this process. The consensus labels and case-specific rationales were subsequently frozen as the evaluator-only Formal Ground Truth before any Formal method execution.

The experts’ consensus process was used only to construct the evaluator oracle. Neither expert tuned the Safety Validator against method outputs, and the finalized oracle could not be queried by any method during execution. Thus, shared semantic rules defined the task, while annotation workflow and runtime information flow remained separated.

Agreement statistics characterize the independent first-pass annotations. Exact risk-class agreement was 93.3%, with quadratic weighted Cohen’s kappa 0.977 [40]. Required-action exact-set agreement was 67.3% and mean Jaccard similarity was 0.912. Forbidden-action set, safety-intervention, and fallback agreement were each 100%, and paraphrase-equivalence agreement was 92.0%. The consensus-adjudicated labels constituted the final evaluation oracle.

### 3.9 Model, provider, and execution configuration

All Formal methods used qwen-plus through the DashScope OpenAI-compatible chat-completions interface. The configured region was cn-beijing, the endpoint host was dashscope.aliyuncs.com, temperature was 0.1, top-p was 0.8, and timeout was 30 seconds. The Qwen model family is described in its technical report [41]. Credentials were loaded from the DASHSCOPE_API_KEY environment variable or a local ignored.env file and were never written to code, Git, research results, or manuscript artifacts.

The provider returned the requested model identifier qwen-plus; no immutable provider-side snapshot identifier, model revision, dated alias, backend version, or deployment identifier was exposed in the retained API metadata. Frozen inputs, retained raw model responses, canonical outputs, provider/configuration metadata, seeds, manifests, and author-generated scientific and analysis code are publicly available in Zenodo under DOI 10.5281/zenodo.22029432, supporting reproduction and audit of the reported analyses from the retained observations. Redistribution-restricted proprietary vendor assets and mappings are excluded and are not required to reproduce the scientific decision and statistical analyses. The public materials do not imply that a future stochastic call to a non-versioned commercial API will reproduce a response bit for bit.

A secret-free connectivity record confirmed that the requested and returned model names matched, the endpoint and region were consistent, structured JSON was returned, token counts were present, and no authentication, billing, or provider error occurred. Formal method order was shuffled independently within each Language Input using seed 20260811. The frozen matrix contained 450 inputs × 3 methods = 1,350 planned runs, one run per input-method pair.

The scientific runtime used Python 3.12.13 on Windows 11. The method implementation used the Python standard library. Formal execution used the frozen source tree and recorded append-only row-level results and raw responses. Failed or unfavorable observations were retained, all planned observations were uniquely indexed, and completed records were not overwritten.

The dispatcher, canonical schema, arbitration interfaces, deterministic Safety Validator, and local Runtime Fallback are architecturally separable from a particular LLM family. Their empirical performance is not model-independent: candidate quality, schema compliance, disagreement, latency, and failure frequency may change across model families or provider updates, and a different model may require prompt/interface adaptation. Any replacement model or provider must therefore be treated as a new frozen configuration and must repeat provider validation, benchmark evaluation, ablation where applicable, stability testing, and boundary checks before the present estimates are transferred.

### 3.10 Endpoints and evaluator

The primary endpoint was Task Success, evaluated from the final canonical semantic action set against adjudicated required and forbidden actions. At the Base level, each method’s score was the mean of its three language-variant outcomes. A strict sensitivity required all three variants of a base to succeed.

The frozen key-secondary family compared SCI-Proposed with EI-MAS on: (1) complete Safety Requirement Satisfaction among 120 safety-required Base Scenarios (360 inputs per method); (2) Failure Recovery among 30 Failure bases (90 inputs per method); and (3) Unsafe Action in an explicit opportunity subset of 35 bases (105 inputs per method). The unsafe subset denominator was never treated as 105 independent scenarios.

Descriptive secondary measures included action-level safety recall and precision, unnecessary safety intervention, routing accuracy, risk-sensitive routing, risk classification, and latency. Latency was measured from recorded host execution intervals. Because one retained EI-MAS observation contained a documented scheduling/infrastructure anomaly, medians, interquartile ranges, 95th percentiles, and a prespecified sensitivity excluding only that anomaly were reported alongside the unmodified primary recorded-data summary.

### 3.11 Statistical analysis

The Base Scenario was the inferential unit. The three language variants were clustered repeated observations. Paired method differences were estimated from Base-level scores with 10,000 Base-cluster bootstrap resamples using seed 20260814; the bootstrap procedure followed standard resampling principles [42]. Exact paired permutation or exact McNemar procedures were used as specified for paired outcomes. The three key-secondary hypotheses were adjusted with Holm’s sequential procedure [43]. Bootstrap confidence intervals and exact paired p values were generated by different prespecified procedures; significance interpretation followed the multiplicity-adjusted paired tests.

Category and Ground-Truth-risk analyses were secondary or exploratory. No post hoc Phase 7 test was added. For HIL proportions and Decision Stability, exact two-sided binomial confidence intervals were used [44]. P values were displayed according to PLOS convention: exact values when p ≥ 0.001 and p < 0.001 when smaller.

### 3.12 Frozen ablation experiment

The ablation experiment compared Full SCI-Proposed with otherwise frozen variants without Risk Assessment, without the Safety Validator, and without Runtime Fallback. One hundred eligible Base Scenarios contributed three variants to each of four conditions, yielding 1,200 rows. The unit, clustered bootstrap, randomization seed 20260812, and Holm family were frozen before analysis. Attempt 1 was preserved but invalid for inference because external quota exhaustion contaminated the intended model-call sequence. Attempt 2 completed 1,200/1,200 rows and was the sole authoritative ablation dataset.

The prespecified component endpoints were Task Success for Risk Assessment, complete Safety Requirement Satisfaction for the Safety Validator, and Failure Recovery for Runtime Fallback. Contribution classification followed the Holm-adjusted tests. Thus, an unadjusted confidence interval excluding zero did not by itself establish a core contribution when the adjusted exact p value did not meet the criterion.

### 3.13 Decision Stability experiment

Thirty frozen Base Scenarios were each executed three new times with Variant A and the full frozen method, producing 90 new runs. Historical Formal Main responses were not reused. Final outputs were canonicalized as evaluator-relevant semantic action sets; raw wording equality was not used. A scenario was stable only when all three repetitions produced exactly the same canonical set. Secondary agreement endpoints covered risk, activated agents, routing, Task Success, Safe Decision, complete Safety Requirement Satisfaction, and fallback activation where applicable. Mean pairwise Jaccard similarity summarized partial set agreement.

### 3.14 Isolated-sandbox HIL validation

HIL was used as a bounded system/integration validation, not as primary causal evidence. HIL commonly supports repeatable testing of automotive electronic-control software against controlled environments [45]. The present platform used one RK3588-family Android 12/API 32 arm64-v8a device, application package com.smartcar.agent, vendor service com.neuedu.canservice/.service.CarService, and an isolated can0 interface using rockchip_canfd at 100000 bit/s.

The frozen HIL boundary prohibited direct application access to can0, hazardous commands, Ground-Truth/evaluator fields, and unversioned mappings. Scientific scoring occurred before the separate HIL interlock and Adapter v1.1. Engineering disposition could not alter Task Success or Safety Requirement Satisfaction. Only versioned allowlisted semantic mappings could proceed to the contained AIDL/vendor sandbox; blocked or unsupported semantics terminated fail-closed.

Before Formal HIL, a separate nonresearch engineering test characterized a fan-level request and verified one controlled level-3 request through the application, containment layer, AIDL, vendor service, and isolated sandbox-CAN evidence chain. This test established one-to-one request-chain transport with duplicate suppression but no protocol acknowledgment or physical blower-actuation claim. Scientific SET_AC_TEMPERATURE remained unsupported and was never converted to fan speed.

Formal HIL selected 30 prespecified Variant A Base Scenarios, six per category, without using Formal, ablation, or stability outcomes for selection. Each was executed once with full SCI-Proposed. End-to-End System Success required a trace-complete unambiguous terminal engineering disposition; it was not equivalent to Scientific Task Success. Safety Enforcement Success applied only where the HIL safety boundary had an enforcement opportunity and was not equivalent to complete scientific Safety Requirement Satisfaction. Gateway Delivery Success required at least one permitted mapped command and was not estimable if the applicable denominator was zero.

This research does not claim conformance with or certification under the ISO 26262 automotive functional-safety lifecycle [46]. The HIL evidence is a contained integration result for the tested platform and interfaces.

### 3.15 Research-integrity and reproducibility controls

Pilot observations were permanently excluded from confirmatory inference, and the method, dataset, Ground Truth, ontology, policy, configuration, and statistical plan were frozen before Formal outcome inspection. Ground Truth entered only the evaluator after a final canonical action set had been produced; the Context Builder rejected evaluator-oracle fields, and no Scenario-ID-specific decision branch was permitted. Planned research rows and raw responses were retained append-only, including unfavorable observations, without silent reruns or overwriting completed records. The same frozen model and provider configuration applied across the compared Formal methods, while randomization, seeds, failure injection, and endpoint definitions were preserved in versioned manifests. Ablation Attempt 1 remained preserved as invalid provenance, whereas the complete Attempt 2 was authoritative. Corrected derived metrics were versioned without changing raw observations. In HIL, scientific scoring was completed before the separate engineering disposition path, which could not rewrite the scientific action set. These controls support reproducibility, provenance, and independent inspection of each prespecified analysis stage, but are not presented as formal verification.

### 3.16 Use of artificial intelligence tools and technologies

Three distinct uses of AI are disclosed. First, qwen-plus, accessed through DashScope, was the experimental model under study and generated the research responses for Single LLM, EI-MAS, SCI-Proposed, ablation, Decision Stability, and Formal HIL scientific decisions under the frozen configuration described above. Second, OpenAI Codex assisted scenario wording, paraphrase generation, and preliminary annotation during dataset preparation. Project logs identify the tool for this role but do not establish the exact historical Codex model version, so no version is inferred. Preliminary AI annotation values were not shown to Reviewer A or Reviewer B during their independent first-pass annotation. The two reviewers independently annotated the scenarios and subsequently resolved disagreements through consensus using frozen annotation criteria before Formal method outcomes existed, as described in Section 3.8. The resulting consensus labels constituted the evaluator-only Formal Ground Truth and were excluded from all method-visible prompts.

Third, OpenAI ChatGPT and OpenAI Codex were used for code drafting and review, audit scripting, document organization, language editing, reference checking, release-package preparation, and Word generation. Reliable project records do not establish exact historic OpenAI model versions for every interaction, so no version was guessed. Human authors directed these uses, inspected code and audit outputs against frozen tests and source artifacts, verified references against bibliographic records, reviewed all manuscript language, and approved every scientific interpretation. These tools affected draft code, auxiliary audit/documentation scripts, manuscript prose, and file generation; OpenAI Codex also affected the dataset-preparation materials disclosed in the preceding paragraph. They did not replace expert adjudication, enter the evaluator-only Ground Truth, alter frozen primary observations or statistical results, authorize silent reruns, or qualify for authorship.

### 3.17 Ethics and operational safety

The experiments used synthetic or research-constructed semantic scenarios and an isolated vehicle sandbox. They did not collect patient data, personal driver behavior, public-road recordings, or physical-vehicle motion outcomes. The HIL procedure prohibited steering, braking, throttle, hazardous body-control commands, and direct application-originated CAN access. Formal HIL generated no permitted mapped command and no direct HIL-originated can0 call.

During revision, the relevant institutional office at Beijing Information Technology College confirmed in writing that the expert-annotation activity did not fall within the scope of human-subject research requiring ethics review. No human research participants were enrolled as study subjects. The two domain experts acted as professional annotators and adjudicators of research-constructed scenarios; their personal, clinical, physiological, or behavioral characteristics were not analyzed as outcomes. They completed independent first-pass annotations and jointly resolved disagreements by consensus before Formal method outcomes were available. Only deidentified expertise descriptors and aggregate agreement statistics are reported. No reviewer names or identifiable comments appear in the manuscript or public archive. Because the activity was outside human-subject research, participant informed-consent procedures were not applicable. This revision-stage scope clarification is not presented as a pre-study ethics approval, retrospective approval, or consent waiver, and no approval or waiver number is claimed.

## 4. Results

### 4.1 Formal Main Task Success

The Formal run completed all 1,350 planned method-input pairs with no missing or duplicate records and no silent reruns. SCI-Proposed succeeded on 360/450 Language Inputs (80.0%), EI-MAS on 169/450 (37.6%), and Single LLM on 155/450 (34.4%). Strict all-three-variant success was 118/150, 51/150, and 47/150 Base Scenarios, respectively (Table 1).

**Table 1:** Formal Main Task Success and Base-level paired effects.

A. Method-level performance			
Method	Successful Language Inputs, n/N (%)		Strict all-three successful Base Scenarios, n/N
SCI-Proposed-v1.0	360/450 (80.0%)		118/150
EI-MAS	169/450 (37.6%)		51/150
Single LLM	155/450 (34.4%)		47/150
**B. Base-level paired effects**			
**Comparison**	**Mean difference**	**95% Base-cluster bootstrap CI**	**Exact paired permutation test**
SCI-Proposed vs EI-MAS	+0.424444	0.348889 to 0.500000	p < 0.001
SCI-Proposed vs Single LLM	+0.455556	0.380000 to 0.531111	p < 0.001

Table note: Base Scenario was the inferential unit (n = 150). Each Base Scenario contributed three clustered Language Inputs per method. Language Inputs were not treated as independent Base Scenarios.

At the Base level, SCI-Proposed exceeded EI-MAS by 0.424444 (95% Base-cluster bootstrap confidence interval [CI], 0.348889 to 0.500000; exact paired permutation p < 0.001) and Single LLM by 0.455556 (95% CI, 0.380000 to 0.531111; p < 0.001). There were 72 positive and 78 tied bases versus EI-MAS, with no negative bases; versus Single LLM, there were 76 positive and 74 tied bases, with no negative bases (Fig 3). Exploratory category results showed no difference in Normal scenarios, where both proposed and EI-MAS means were 1.000, and larger differences in Fatigue, Diagnosis, Compound, and Failure categories. These subgroup findings were not promoted to new confirmatory claims.

**Fig 3:**
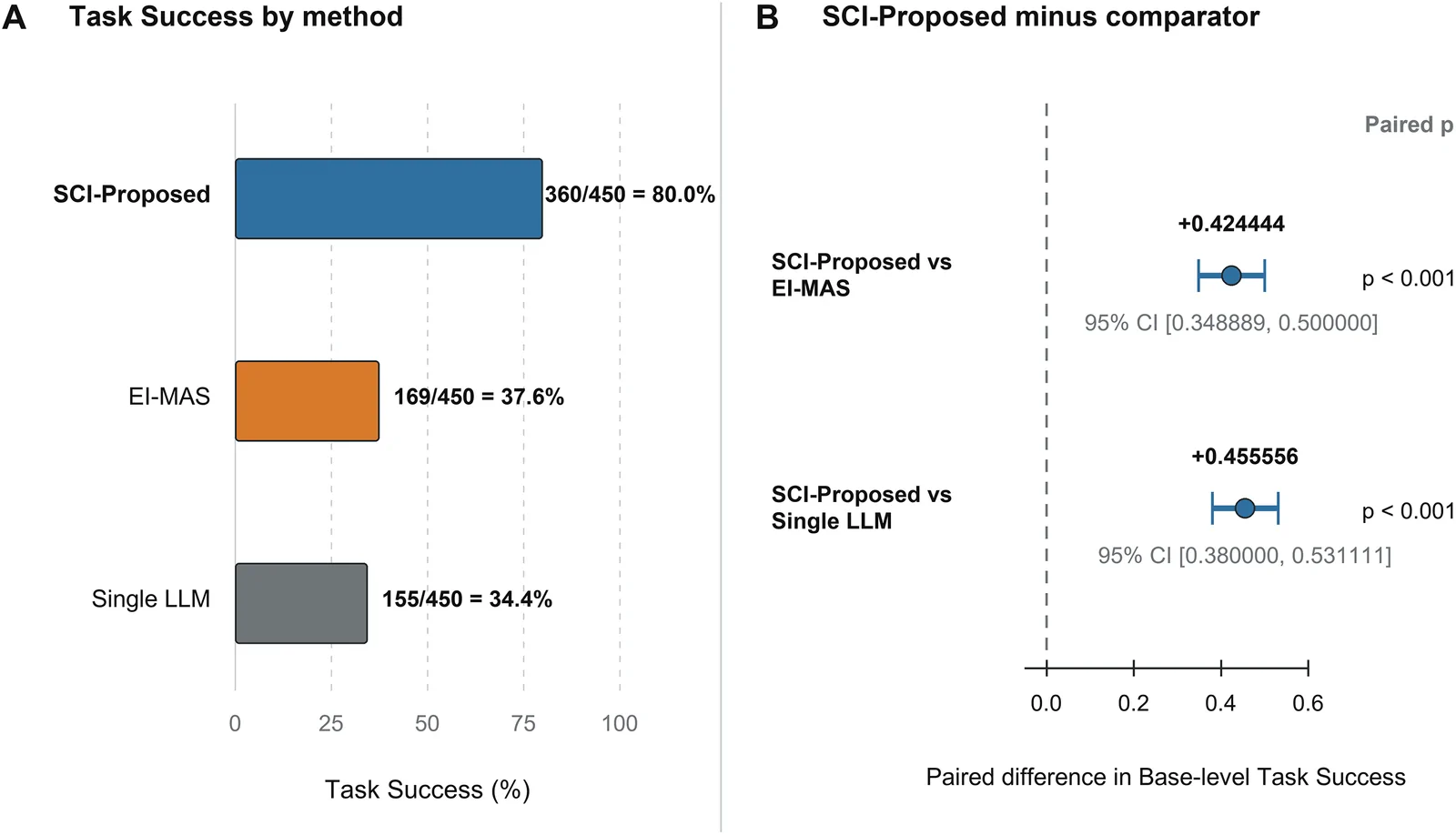
Formal Main Task Success and Base-level paired effects. (A) Descriptive successful Language Inputs were 360/450 (80.0%) for SCI-Proposed, 169/450 (37.6%) for EI-MAS, and 155/450 (34.4%) for Single LLM. (B) SCI-Proposed minus EI-MAS was + 0.424444 (95% Base-cluster bootstrap CI [0.348889, 0.500000], exact paired permutation test, p < 0.001), and SCI-Proposed minus Single LLM was + 0.455556 (95% CI [0.380000, 0.531111], exact paired permutation test, p < 0.001). Base Scenario was the inferential unit; the three language variants were clustered repeated observations rather than independent bases.

### 4.2 Safety Requirement Satisfaction and failure recovery

Among 120 safety-required bases, complete Safety Requirement Satisfaction was 316/360 inputs (87.8%) for SCI-Proposed and 101/360 (28.1%) for EI-MAS. The paired difference was 0.597222 (95% Base-cluster bootstrap CI, 0.513889 to 0.680556; Holm-adjusted p < 0.001). The corresponding Single LLM descriptive count was 91/360 (25.3%).

Failure Recovery was 33/90 (36.7%) for SCI-Proposed and 0/90 for EI-MAS across 30 Failure Base Scenarios. The paired difference was 0.366667 (95% CI, 0.200000 to 0.533333; Holm-adjusted p = 0.00195). Recovery was heterogeneous by frozen fault class: SCI-Proposed recovered 9/9 API failures, 12/12 timeouts, 6/12 unavailable-agent inputs, and 6/12 malformed-output inputs, but recovered none of the represented agent conflicts, contradictory states, invalid execution requests, invalid semantic actions, or model-override cases under the endpoint definition. This result supports recovery only for the represented conditions, not arbitrary failures.

In the unsafe-opportunity subset, SCI-Proposed produced unsafe actions in 0/105 Language Inputs and EI-MAS in 11/105. These inputs arose from 35 independent Base Scenarios. Although the Base-cluster bootstrap CI for the difference excluded zero, the prespecified Base-paired Holm-adjusted p value was 0.0625. Therefore, the unsafe-action comparison was not statistically significant under the frozen testing procedure (Table 2).

**Table 2:** Formal safety, failure-recovery, and action-level safety results.

Complete Safety Requirement Satisfaction	120 safety-required Base Scenarios; 360 Language Inputs/method	316/360 (87.8%)	101/360 (28.1%)	+59.7 percentage points (95% Base-cluster bootstrap CI 51.4 to 68.1 pp)	p<0.001
Failure Recovery	30 Failure Base Scenarios; 90 Language Inputs/method	33/90 (36.7%)	0/90 (0.0%)	+36.7 percentage points (95% CI 20.0 to 53.3 pp)	p=0.00195
Unsafe Action within opportunity subset	35 Base Scenarios; 105 Language Inputs/method	0/105 (0.0%)	11/105 (10.5%)	−10.5 percentage points (95% Base-cluster bootstrap CI −21.0 to −1.9 pp)	p=0.0625
**B. Descriptive action-level safety metrics**					
SCI-Proposed-v1.0	874/927 (94.3%)	874/1,226 (71.3%)	0/90	0/450	0/105
EI-MAS	425/927 (45.8%)	425/477 (89.1%)	0/90	11/450	11/105
Single LLM	408/927 (44.0%)	408/444 (91.9%)	0/90	11/450	11/105

Table note: The 105 Language Inputs arise from 35 independent Base Scenarios. The bootstrap CI and the prespecified exact paired/Holm test use different inferential procedures. Confirmatory significance follows the prespecified multiplicity-adjusted paired test; therefore the unsafe-action comparison is not interpreted as statistically significant.

The three prespecified safety and recovery results are summarized visually in Fig 4.

**Fig 4:**
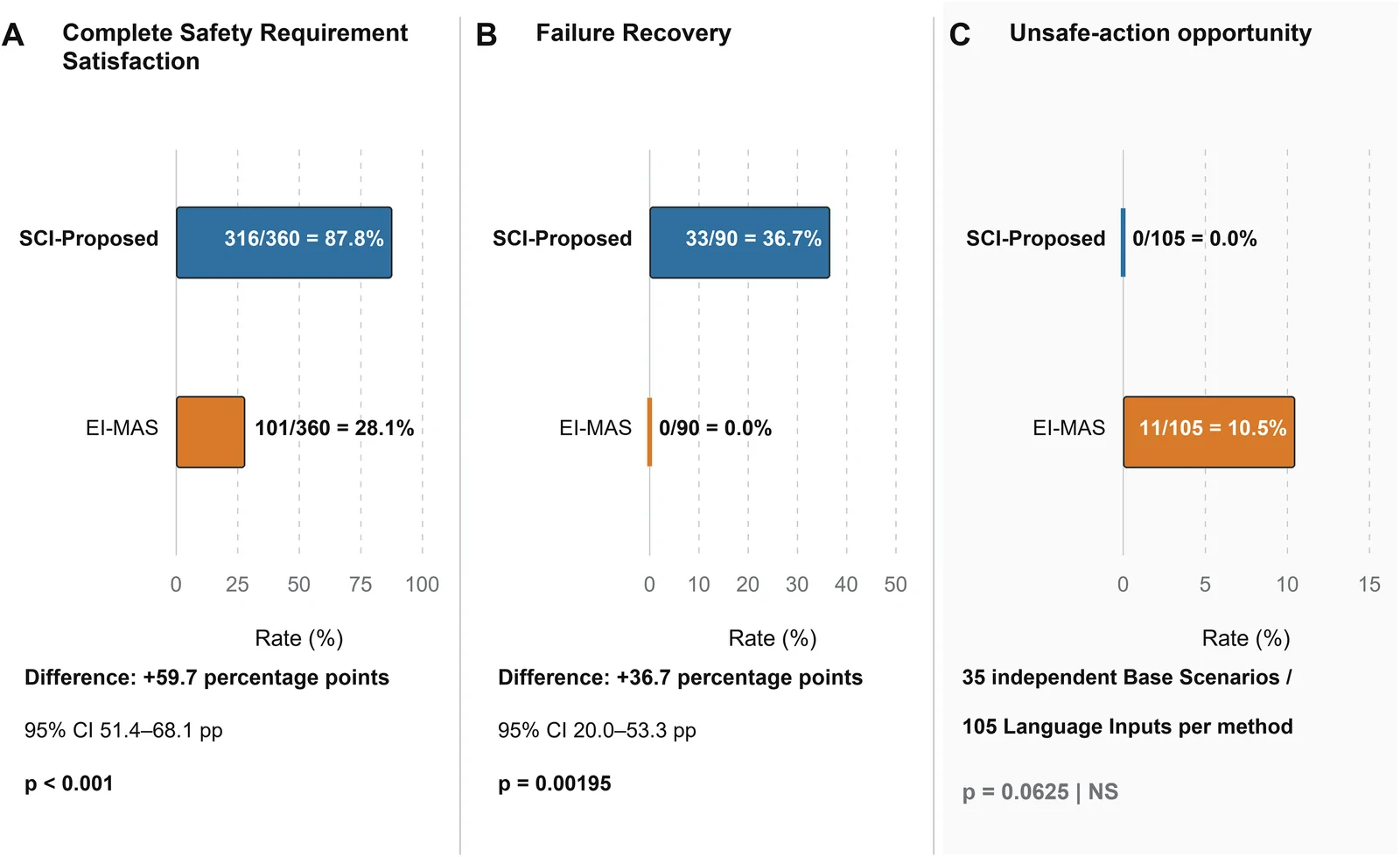
Safety-requirement satisfaction, failure recovery, and unsafe-action opportunities. (A) Complete Safety Requirement Satisfaction was 316/360 (87.8%) for SCI-Proposed and 101/360 (28.1%) for EI-MAS; the paired Base-level difference was 59.7 percentage points (95% Base-cluster bootstrap CI 51.4–68.1 percentage points, Holm-adjusted p < 0.001). (B) Failure Recovery was 33/90 (36.7%) versus 0/90 (0.0%); the paired difference was 36.7 percentage points (95% CI 20.0–53.3 percentage points, Holm-adjusted p = 0.00195) across 30 Failure Base Scenarios. (C) Unsafe actions occurred in 0/105 versus 11/105 opportunity Language Inputs. The 105 unsafe-opportunity Language Inputs arise from 35 independent Base Scenarios. The Base-paired Holm-adjusted p value was 0.0625; therefore this comparison is labeled non-significant and does not support a statistically significant unsafe-action reduction claim.

Complete scenario-level satisfaction and action-level micro measures captured distinct properties. SCI-Proposed action-level recall was 874/927 (94.3%) and precision was 874/1,226 (71.3%). EI-MAS recall and precision were 425/927 (45.8%) and 425/477 (89.1%); Single LLM values were 408/927 (44.0%) and 408/444 (91.9%). No method generated an unnecessary safety intervention in the 90 non-required inputs. The lower precision for SCI-Proposed reflects additional non-required actions under the micro definition and should not be relabeled as unsafe-action rate.

### 4.3 Component ablation

Attempt 2 completed all 1,200 planned rows and preserved the frozen order and component definitions. Removing Risk Assessment reduced Task Success from 0.6967 to 0.6567, a difference of 0.0400 (95% Base-cluster bootstrap CI, 0.0067 to 0.0800). However, the exact and Holm-adjusted p value was 0.125; Risk Assessment was therefore classified as supporting architecture rather than a core causal contribution.

Removing the Safety Validator reduced complete Safety Requirement Satisfaction from 0.8500 to 0.5733, a difference of 0.2767 (95% CI, 0.2000 to 0.3600; Holm-adjusted p < 0.001). Removing Runtime Fallback reduced Failure Recovery from 0.3667 to 0, a difference of 0.3667 (95% CI, 0.2000 to 0.5333; Holm-adjusted p = 0.00195). These two components met the frozen criterion for core independent contributions (Table 3).

**Table 3:** Component effects in the frozen ablation experiment.

Component ablated	Prespecified endpoint	Full	Ablated	Full minus ablated (95% Base-cluster bootstrap CI)	Holm-adjusted p	Contribution classification
Risk Assessment	Task Success	0.6967	0.6567	+0.0400 (0.0067 to 0.0800)	p = 0.125	Supporting
Safety Validator	Safety Requirement Satisfaction	0.8500	0.5733	+0.2767 (0.2000 to 0.3600)	p < 0.001	Core
Runtime Fallback	Failure Recovery	0.3667	0.0000	+0.3667 (0.2000 to 0.5333)	p = 0.00195	Core

Table notes: Values in the Full and Ablated columns are proportions. Base Scenario was the statistical unit; language variants were clustered within Base Scenario. Attempt 2 is authoritative; Attempt 1 was retained for provenance but excluded from inference. Holm adjustment covers exactly the three prespecified component hypotheses. Bootstrap confidence intervals and exact paired Holm-adjusted p values derive from different prespecified inferential procedures; contribution classification follows the multiplicity-adjusted tests.

The component effects and their locked contribution classifications are summarized visually in Fig 5.

**Fig 5:**
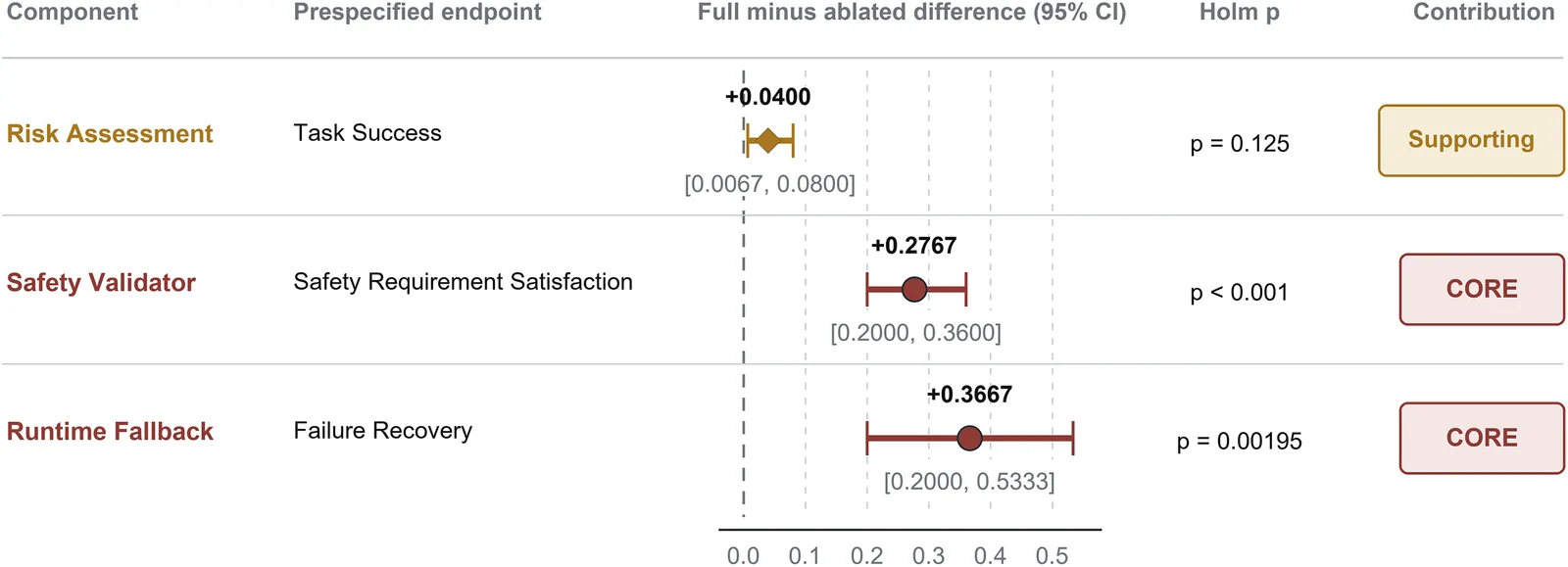
Component-level effects in the frozen ablation experiment. Full-minus-ablated effects were +0.0400 for Risk Assessment on Task Success (95% Base-cluster bootstrap CI [0.0067, 0.0800], Holm-adjusted p = 0.125), + 0.2767 for the Safety Validator on complete Safety Requirement Satisfaction (95% CI [0.2000, 0.3600], p < 0.001), and +0.3667 for Runtime Fallback on Failure Recovery (95% CI [0.2000, 0.5333], p = 0.00195). Holm-adjusted p values correspond to the prespecified three-hypothesis ablation family. Safety Validator and Runtime Fallback are locked as core contributions; Risk Assessment remains supporting architecture and is not marked significant under the prespecified family. Attempt 2 is authoritative; Attempt 1 is excluded from inference.

### 4.4 Decision Stability

Across three new executions per selected base, 27/30 scenarios produced exactly the same canonical action set in all repetitions (90.0%; exact 95% CI, 73.5% to 97.9%). Mean pairwise action-set Jaccard similarity was 0.9830. Risk, activated-agent set, and routing agreement were each 30/30. Task Success agreement was 29/30, Safe Decision agreement was 30/30, corrected complete Safety Requirement Satisfaction agreement was 29/30, and fallback activation agreement was 6/6 applicable Failure scenarios (Table 4).

**Table 4:** Decision stability across three repeated executions.

Endpoint	Result	Definition/ applicable set
Exact 3-of-3 canonical action-set stability	27/30 (90.0%; exact 95% CI 73.5%−97.9%)	Base Scenario
Mean pairwise action-set Jaccard	0.9830	Mean across 30 Base Scenarios
Risk prediction agreement	30/30	All three repetitions agree
Activated-agent-set agreement	30/30	All three repetitions agree
Routing agreement	30/30	All three repetitions agree
Task Success agreement	29/30	All three repetitions agree
Safe Decision agreement	30/30	All three repetitions agree
Complete Safety Requirement Satisfaction agreement	29/30	All three repetitions agree
Fallback activation agreement	6/6	Applicable Failure Base Scenarios

Table note: Thirty frozen Base Scenarios were each executed in three new repetitions (90 runs). Agreement was evaluated on canonical semantic outputs rather than raw natural-language equality.

The three unstable scenarios were retained. One involved non-safety semantic variation, one involved safety-related safe-stop variation while mandatory actions remained satisfied in all repetitions, and one involved mandatory safe-stop instability with complete-satisfaction values false/true/false. Thus, high average set similarity did not eliminate scenario-level instability, and the 27/30 exact endpoint—not 30/30—is the primary repeatability result.

### 4.5 Formal HIL validation

Formal HIL completed the prespecified 30 cases without substitutions, silent reruns, deleted unfavorable cases, Ground-Truth leakage, or changes to frozen scientific identities. Scientific Task Success was 25/30: 6/6 Normal, 6/6 Fatigue, 4/6 Diagnosis, 5/6 Compound, and 4/6 Failure. Safe Decision was 30/30, complete Safety Requirement Satisfaction was 29/30, and exact risk agreement was 28/30.

All cases reached a trace-complete terminal disposition, giving End-to-End System Success of 30/30 (exact 95% CI, 88.4% to 100.0%). Safety Enforcement Success was 7/7 applicable cases (95% CI, 59.0% to 100.0%). Runtime Recovery was 4/6 (95% CI, 22.3% to 95.7%): 0/1 for agent conflict, 1/2 for unavailable agent, and 1/1 each for API failure, timeout, and malformed output.

No Formal HIL case contained a permitted mapped command. Gateway Delivery Success was therefore not estimable (applicable N = 0), rather than 0% or 100%. Two scientifically valid SET_AC_TEMPERATURE actions terminated as UNSUPPORTED_VENDOR_SEMANTIC_MAPPING. There were zero unintended deliveries, zero Formal engineering fan-control calls, and zero direct HIL-originated can0 calls (Table 5).

**Table 5:** Formal isolated-sandbox HIL validation endpoints.

Endpoint	Success/ applicable N	Result	95% CI/ interpretation
Scientific Task Success	25/30	83.3%	Pre-transport scientific endpoint
Safe Decision	30/30	100.0%	Not equivalent to complete safety satisfaction
Complete Safety Requirement Satisfaction	29/30	96.7%	Complete scientific requirement
Exact risk-prediction agreement	28/30	93.3%	Predicted vs frozen Ground Truth risk
End-to-End System Success	30/30	100.0%	Exact 95% CI 88.4%−100.0%; trace-complete terminal disposition
Safety Enforcement Success	7/7	100.0%	Exact 95% CI 59.0%−100.0%
Runtime Recovery	4/6	66.7%	Exact 95% CI 22.3%−95.7%
Gateway Delivery Success	N = 0	Not estimable	0 applicable scenarios; 0 permitted mapped commands

Table notes: End-to-End System Success is not equivalent to Scientific Task Success. Safety Enforcement Success is not equivalent to Complete Safety Requirement Satisfaction. Gateway Delivery Success is not estimable when N = 0. Two scientific SET_AC_TEMPERATURE actions terminated as UNSUPPORTED_VENDOR_SEMANTIC_MAPPING. There were 0 unintended deliveries, 0 Formal engineering fan-control calls, and 0 direct HIL-originated can0 calls.

The scientific-to-engineering boundary and Formal HIL dispositions are summarized in Fig 6.

**Fig 6:**
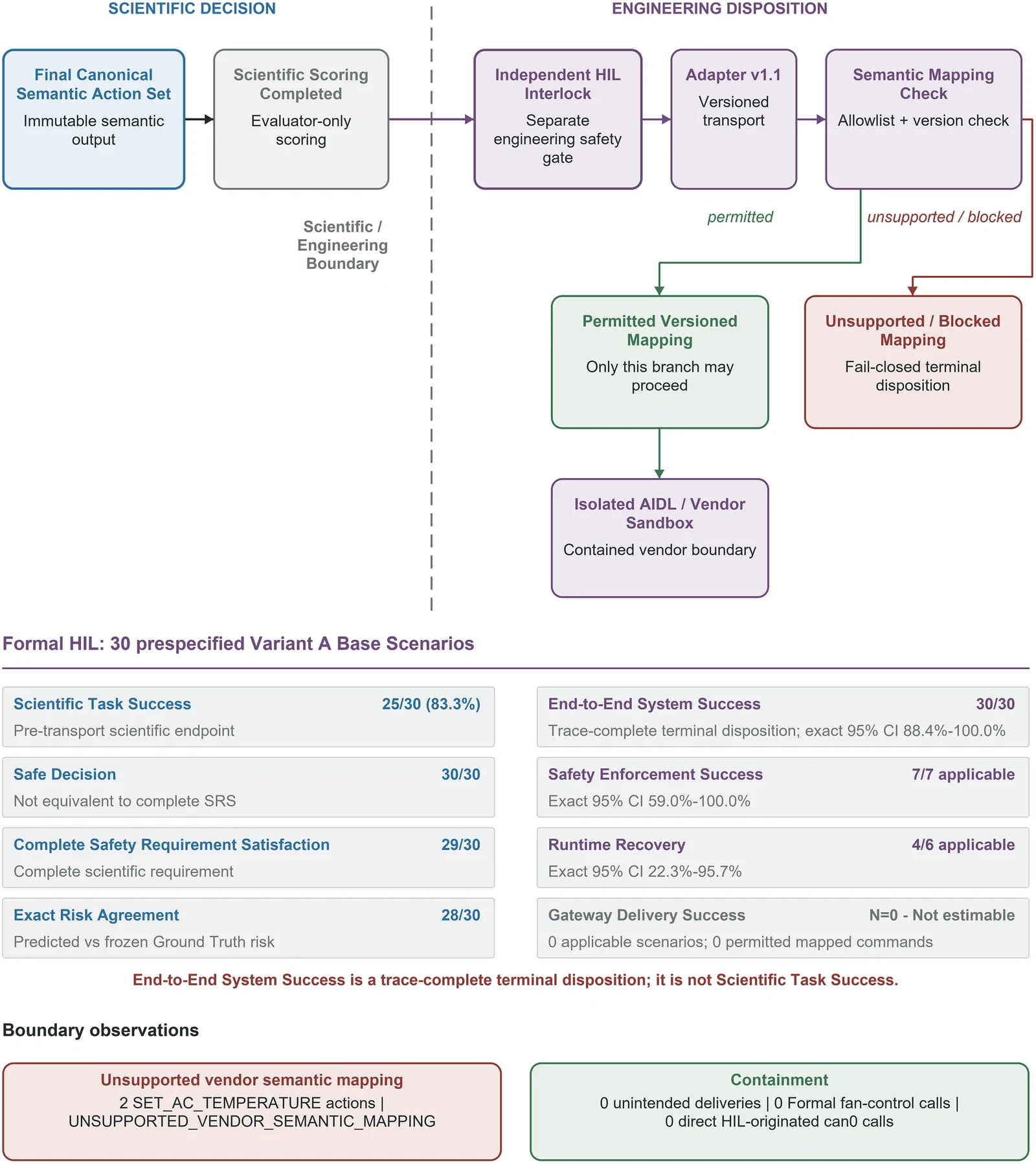
Isolated-sandbox HIL validation and preservation of the scientific decision boundary. The final canonical semantic action set is scientifically scored before the separate engineering path consisting of the independent HIL interlock, versioned Adapter v1.1, semantic mapping check, and contained AIDL/vendor sandbox. Only permitted versioned mappings may reach the vendor sandbox; unsupported or blocked mappings terminate fail-closed. Across 30 prespecified Variant A Formal HIL cases, Scientific Task Success was 25/30 (83.3%), Safe Decision was 30/30, complete Safety Requirement Satisfaction was 29/30, and exact risk agreement was 28/30. End-to-End System Success was 30/30 (exact 95% CI 88.4%−100.0%), Safety Enforcement Success was 7/7 applicable (95% CI 59.0%−100.0%), and Runtime Recovery was 4/6 applicable (95% CI 22.3%−95.7%). End-to-End System Success does not equal Scientific Task Success; Gateway Delivery was not estimable because N = 0. Two SET_AC_TEMPERATURE actions terminated as UNSUPPORTED_VENDOR_SEMANTIC_MAPPING. There were zero unintended deliveries, zero Formal fan-control calls, and zero direct HIL-originated can0 calls. Nonresearch engineering characterization is presented separately in S1 Fig and is not part of the Formal scientific actions.

### 4.6 Latency

Formal Main median recorded latency was 2,094.318 ms for SCI-Proposed, 2,092.256 ms for EI-MAS, and 2,064.568 ms for Single LLM. SCI-Proposed 95th-percentile latency was 8,866.778 ms. The EI-MAS recorded-data mean was heavily contaminated by one retained scheduling/infrastructure anomaly; excluding only that prespecified observation in sensitivity analysis changed its overall mean from 24,894.444 ms to 2,723.230 ms, while the median changed from 2,092.256 ms to 2,081.559 ms. Latency was descriptive and not used to define a component contribution.

Decision Stability latency across 90 runs had a mean of 3,882.119 ms, median 1,952.445 ms, interquartile range 3,709.623 ms, and 95th percentile 10,878.043 ms. Formal HIL scientific-decision latency across 30 cases had a mean of 3,445.070 ms, median 2,456.369 ms, and 95th percentile 10,280.141 ms. The engineering disposition gate mean was 0.004 ms. Device/gateway latency was not applicable because no permitted mapped command existed; host and device wall clocks were retained only as provenance and were not cross-subtracted.

## 5. Discussion

### 5.1 Principal findings

This study provides convergent but non-interchangeable evidence for a bounded semantic decision architecture. In the frozen Formal comparison, SCI-Proposed substantially improved Task Success relative to both comparators. The improvement was consistent at the Base-scenario level and persisted under the strict all-three-variant sensitivity. Complete Safety Requirement Satisfaction was also higher than EI-MAS, and the ablation study attributed independent effects to the deterministic Safety Validator and local Runtime Fallback. These two components—not Risk Assessment—constitute the core supported contributions.

The results reinforce a general systems lesson from runtime-assurance research: performance generation and safety enforcement should be evaluated as distinct functions [25–36]. The generative agents proposed semantic actions, but a deterministic validator enforced frozen constraints and contextual requirements. When represented runtime failures raised exceptions, the fallback used local rules rather than asking the failed provider path to repair itself. The architecture did not assume that an LLM could validate its own output or that agent consensus implied safety.

### 5.2 Independent Safety Validation

Prior systems have investigated LLM reasoning for autonomous-driving plans, multimodal closed-loop control, multi-agent driving interaction, and cooperative merging [7–17]. SanDRA further demonstrates how an external reachability-analysis layer can remove unsafe candidate driving actions [18]. SCI-Proposed is complementary rather than directly competitive with those systems. It operates one abstraction level away from motion control and evaluates cockpit, warning, navigation-intent, diagnostic, and recovery semantics. This narrower scope enabled strict separation of a method-visible context, a frozen semantic ontology, an evaluator-only oracle, and an engineering transport boundary.

The distinction matters for interpretation. An action such as NAVIGATE_TO_SAFE_STOP expresses a semantic safety intent; it is not a trajectory, verified reachable set, or command to steering and braking actuators. Similarly, a correct semantic action does not prove that a downstream navigation or control subsystem can execute it safely. The study therefore supports a semantic decision layer that could be integrated with other verified subsystems, not an autonomous-driving stack.

Complete Safety Requirement Satisfaction was an all-required-actions scenario endpoint, whereas micro recall counted action instances and micro precision penalized additional actions. SCI-Proposed combined high micro recall with lower precision than the comparators because it more often added contextual safety actions. These measures should not be conflated with unsafe action occurrence.

The numerical pattern reinforces that distinction. SCI-Proposed achieved 94.3% action-level recall and 71.3% precision. Ground-Truth Required Actions represented a minimum necessary set, so a non-required but non-forbidden contextual action lowered precision without necessarily making the decision unsafe. No method generated an unnecessary safety intervention in the 90 inputs where such an intervention was not required. In the exploratory Normal category, SCI-Proposed and EI-MAS both had mean Task Success of 1.000. Taken together, these observations do not support an explanation based on blanket rejection of ordinary requests or indiscriminate safety escalation; the principal safety evidence instead comes from complete requirement satisfaction and the prespecified Safety Validator ablation. Future comparisons should continue to report completeness, precision, and unsafe occurrence as distinct quantities.

The unsafe-opportunity result also requires restraint. The descriptive counts favored SCI-Proposed (0/105 versus 11/105), but the 105 inputs represented only 35 independent bases. Under the prespecified paired test and Holm family, p = 0.0625. It would therefore be incorrect to claim a statistically significant reduction or universal elimination of unsafe actions. A larger independently sampled set of unsafe opportunities is needed to estimate that effect with greater precision.

### 5.3 Runtime assurance addresses a distinct failure mode

Runtime recovery was similarly bounded. Formal Main recovery concentrated in API failure, timeout, unavailable-agent, and malformed-output cases. Other represented classes had no recovery under the endpoint definition. Formal HIL recovered four of six selected failures, with one failed agent-conflict case and one failed unavailable-agent case. Unknown, simultaneous, compound, and out-of-ontology failures were not tested, and no universal safe-default performance was estimated. The system should therefore be described as runtime-assured only for the specified local failure handling and represented conditions, not as universally fault tolerant.

### 5.4 Multi-agent coordination and component hierarchy

The causal interpretation must remain within the frozen endpoints. Removing the Safety Validator decreased complete safety satisfaction by 0.2767, and removing Runtime Fallback decreased represented-failure recovery by 0.3667. By contrast, the risk ablation produced a small Task Success difference whose bootstrap interval excluded zero but whose prespecified Holm-adjusted exact p value was 0.125. Accordingly, risk-informed coordination can be described as useful supporting architecture, but not as a dominant or independently confirmed core mechanism. The evidence therefore supports a hierarchy in which generative specialization and risk-informed routing organize candidate decisions, while the independently tested Safety Validator and Runtime Fallback provide the core assurance contributions.

### 5.5 Repeatability and engineering-boundary preservation

Canonicalization separated meaningful action-set variation from superficial text variation. Exact three-of-three stability was 27/30 and mean pairwise Jaccard similarity was 0.9830. These results show strong repeatability in the selected sample, but the three unstable cases remain scientifically important. One changed complete Safety Requirement Satisfaction across repetitions. Thus, stable routing and risk classification did not guarantee an identical final action set.

Formal HIL demonstrated containment and trace completion, not physical control success. The 30/30 End-to-End System Success endpoint meant that every case had an unambiguous terminal scientific-to-engineering disposition. It did not mean that every scientific task succeeded; Scientific Task Success was 25/30. Likewise, 7/7 Safety Enforcement Success referred only to applicable HIL enforcement opportunities and was distinct from 29/30 complete scientific safety satisfaction.

Gateway Delivery Success was not estimable because no Formal HIL case contained a permitted mapped command. Reporting 0% would imply failed applicable deliveries, while reporting 100% would imply successful applicable deliveries; neither occurred. The two temperature actions correctly terminated as unsupported mappings, preserving rather than silently translating the scientific semantics.

The separate engineering fan characterization supplied limited transport evidence: one controlled request reached the vendor service and isolated sandbox evidence chain, duplicate input was suppressed, and prohibited cases failed closed. The terminal status explicitly lacked protocol acknowledgment. No inference about physical blower actuation, vehicle effect, or temperature-to-fan equivalence follows. This evidence is appropriately placed in Supporting Information rather than treated as a Formal scientific endpoint.

The interpretive value of HIL therefore lies in boundary preservation. Ground Truth never entered method-visible prompts, scientific scoring preceded transport, and the HIL interlock, containment guard, and Adapter could not rewrite the canonical action set. This separation made it possible to report terminal system disposition without inflating scientific performance or converting a semantic result into a physical-actuation claim.

A validated semantic action should be interpreted as an upstream contract, not as a direct execution authorization. Before such an action can influence physical behavior, a practical vehicle stack would additionally require a versioned semantic-to-planning mapping, planner-level operational constraints, control-level safety and runtime assurance, protocol and actuator acknowledgement, execution feedback, vehicle-state verification, fault monitoring, and lifecycle/configuration controls. None of those downstream assurances was established by the present HIL study. Future HIL work should prospectively include permitted nonhazardous mappings, acknowledged transport, state-feedback verification, additional devices/interfaces, and larger independently selected samples while retaining the same scientific/engineering separation.

### 5.6 Implications and limitations

A canonical semantic action boundary makes independent policy checks, evaluator isolation, versioned adapters, and fail-closed dispositions testable without converting model text directly into transport requests.

External generalizability is limited by an author-constructed synthetic benchmark, research-constructed comparators, one model/provider configuration, and language and structured-state distributions that may not reflect naturalistic fleet data, sensor uncertainty, or other operational design domains. The comparators were controls within a common semantic interface rather than exhaustive state-of-the-art or production systems, so the results do not establish general multi-agent superiority. Independently authored datasets, external sites, and additional vehicle contexts are required.

Comparator and fault coverage were bounded: not every agent design was represented, recovery applied only to prespecified single primary injected failure classes and targets, and stability used 30 bases with three repetitions. Unknown, simultaneous, compound, and out-of-ontology failures were not validated. The comparison was architecture-matched rather than inference-budget-matched; published autonomous-driving stacks were not directly benchmarked because their inputs, outputs, and control abstractions differ from the frozen semantic-decision task.

Ground Truth was based on two independent domain-review streams followed by consensus adjudication of disagreement cases using frozen annotation criteria. The Safety Validator and Ground Truth intentionally used the same frozen semantic policy domain because policy compliance was the endpoint, while Scenario IDs, oracle values, draft annotations, and method outputs remained unavailable to the Validator. This specification-level alignment defines the compliance target, while the independent review/freeze and separated runtime information flow provide procedural and informational independence. Accordingly, this endpoint measures enforcement within the prespecified specification rather than external safety validity. Agreement was high for risk and safety-critical fields, whereas required-action exact-set agreement was lower, reflecting greater judgment in defining minimum necessary semantic actions. Original completed first-pass workbooks were not retained in the final archive; reconstructed preservation views and the adjudicated candidate record are therefore provided for provenance. The unsafe-opportunity sample was 35 bases and not significant (p = 0.0625).

HIL used one device, one isolated vendor sandbox, and 30 prespecified cases. No Formal case contained a permitted mapped command, so Gateway Delivery Success was not estimable (N = 0), and no physical-actuator acknowledgement or vehicle-motion outcome existed. The HIL results establish trace completion and fail-closed preservation of the tested semantic-to-engineering boundary; they do not establish permitted-command delivery reliability, physical execution, public-road safety, production readiness, or certification. Proprietary vendor assets and mappings cannot be released. Author-generated data and scientific code are publicly deposited in Zenodo under DOI 10.5281/zenodo.22029432; redistribution-restricted proprietary vendor materials remain excluded.

The provider exposed no immutable qwen-plus snapshot, so the retained responses and offline analyses are reproducible from the archive, but bitwise regeneration of responses through the commercial API is not guaranteed. Threshold sensitivity was not tested, and the prespecified risk anchors may require recalibration across populations, vehicles, sensors, and environments. Future work should test additional model families and providers under newly frozen configurations, independently authored datasets, a larger unsafe-opportunity sample, broader and compound fault classes, threshold sensitivity, more stability repetitions, and prespecified HIL cases with permitted nonhazardous mappings, protocol acknowledgement, execution feedback, and state verification.

## 6. Conclusions

SCI-Proposed-v1.0 combined lightweight LLM multi-agent semantic reasoning with an independent deterministic Safety Validator and conditional local Runtime Fallback. In a frozen 150-base, 450-input comparison, it improved Task Success and complete Safety Requirement Satisfaction relative to the tested baselines. Frozen ablation identified the Safety Validator and Runtime Fallback as core supported components for the represented conditions, while Risk Assessment remained supporting architecture. Repeated executions showed 27/30 exact canonical action-set stability, and isolated-sandbox HIL preserved a traceable fail-closed boundary with 29/30 complete safety satisfaction and 4/6 runtime recovery.

The main mechanistic result was therefore not that adding more generative agents was sufficient. The strongest evidence favored assurance mechanisms placed outside ordinary model generation: the Safety Validator evaluated completed canonical actions against a frozen policy, whereas Runtime Fallback used local facts only after represented failures interrupted the normal path. Keeping these roles separate made their effects independently testable and prevented successful provider calls, safety-policy compliance, runtime recovery, and engineering disposition from collapsing into one undifferentiated success measure. Decision Stability further showed that high average semantic similarity does not guarantee identical scenario-level outputs, and HIL showed that a trace-complete engineering disposition is not equivalent to Scientific Task Success.

These findings support layered assurance for bounded semantic vehicle decisions under the tested qwen-plus/DashScope configuration, frozen semantic policy, controlled benchmark, and represented failure classes. They do not establish autonomous-driving capability, broader vehicle-system safety, arbitrary- or compound-fault recovery, physical execution reliability, public-road readiness, production readiness, or functional-safety certification. The HIL evidence is limited to traceable boundary preservation in one isolated platform, and the commercial-model results require revalidation after model/provider changes. Further external, multi-model, independently authored, larger-sample, threshold-sensitivity, and protocol-acknowledged validation is required before broader claims can be considered. Within this bounded scope, generative reasoning, explicit semantic-policy enforcement, and represented-failure recovery can be architecturally separated and evaluated on their own prespecified endpoints.

## Supporting information

S1 Fig Nonresearch HIL engineering characterization of the versioned fan-level request chain. This engineering-only test was separate from the Formal scientific actions and scientific scoring. One controlled level-3 request produced one AIDL call, one vendor receipt, and one ECHO-collapsed logical sandbox-CAN group. Duplicate input [3,3] produced one unique delivery and one suppressed duplicate, while prohibited checks failed closed. The characterization does not establish a SET_AC_TEMPERATURE-to-fan-speed semantic equivalence, physical blower actuation, or protocol acknowledgment.
(TIF)

S1 Table Category-level Task Success and failure-class Runtime Recovery in Formal HIL. Panel A reports Scientific Task Success for Normal (6/6), Fatigue (6/6), Diagnosis (4/6), Compound (5/6), and Failure (4/6) scenarios. Panel B reports Runtime Recovery for AGENT_CONFLICT (0/1), AGENT_UNAVAILABLE (1/2), API_FAILURE (1/1), LLM_TIMEOUT (1/1), and MALFORMED_OUTPUT (1/1). Small-cell percentages are intentionally omitted.
(DOCX)
