# Supplementary material for: Safety-constrained and runtime-assured LLM multi-agent decision making for intelligent vehicle systems
Source: PLoS One. 2026 Oct 30;21(10):e0357928. doi: 10.1371/journal.pone.0357928 (PMC13722150; doi:10.1371/journal.pone.0357928)
Supplement: Supplementary file 1 [file pone.0357928.s002.docx]

**S1 Table. Category-level Task Success and failure-class Runtime Recovery in Formal HIL.**

**A. Scientific Task Success by category**

| **Category** | **Scientific Task Success, n/N** |
| --- | --- |
| Normal | 6/6 |
| Fatigue | 6/6 |
| Diagnosis | 4/6 |
| Compound | 5/6 |
| Failure | 4/6 |

**B. Runtime Recovery by represented failure class**

| **Represented failure class** | **Runtime Recovery, n/N** |
| --- | --- |
| AGENT_CONFLICT | 0/1 |
| AGENT_UNAVAILABLE | 1/2 |
| API_FAILURE | 1/1 |
| LLM_TIMEOUT | 1/1 |
| MALFORMED_OUTPUT | 1/1 |
