# Supplementary material for: Safety-constrained and runtime-assured LLM multi-agent decision making for intelligent vehicle systems
Source: PLoS One. 2026 Oct 30;21(10):e0357928. doi: 10.1371/journal.pone.0357928 (PMC13722150; doi:10.1371/journal.pone.0357928)
Supplement: Supplementary file 2 [file pone.0357928.s004.docx]

**Response to the Academic Editor and Reviewers**
Manuscript ID: PONE-D-26-42132
Safety-constrained and runtime-assured LLM multi-agent decision making for intelligent vehicle systems

Dear Academic Editor and Reviewers,

We thank you for the careful and constructive evaluation. We revised the manuscript point by point while preserving the frozen scientific record. No experiment was rerun; no raw observation, denominator, percentage, p value, confidence interval, table, figure, Supporting Information item, Ground Truth label, or statistical endpoint was changed. New text clarifies the policy/ontology construction and extension process, threshold interpretation, failure-coverage boundary, model/provider limitations, Ground Truth–Validator relationship, semantic-to-control boundary, HIL interpretation, AI disclosure, ethics scope, and conclusions. Two reviewer-recommended references were verified and added for background context. Revised text locations are identified by section and paragraph beginning rather than potentially unstable page/line numbers.

JOURNAL REQUIREMENTS

====================

**Journal Requirements — Point 1**

**Comment (verbatim):** *Please ensure that your manuscript meets PLOS ONE's style requirements, including those for file naming. The PLOS ONE style templates can be found at https://journals.plos.org/plosone/s/file?id=wjVg/PLOSOne_formatting_sample_main_body.pdf and https://journals.plos.org/plosone/s/file?id=ba62/PLOSOne_formatting_sample_title_authors_affiliations.pdf*

**Response:** We revised the manuscript and prepared separate clean and visibly marked Word files with simple journal-facing filenames. The scientific tables, figures, Supporting Information, and numerical results were left unchanged. We also performed a separate render and formatting audit.

**Changes in the revised manuscript:** Whole manuscript and revision package.

**Journal Requirements — Point 2**

**Comment (verbatim):** *We note that you have declared the use of generative AI in the preparation of your manuscript. Please note that contributions by artificial intelligence (AI) tools and technologies to a study or to an article’s contents must be clearly reported in a dedicated section of the Methods, or in the Acknowledgements section for article types lacking a Methods section. This section should include the name(s) of any tools used, a description of how the authors used the tool(s) and evaluated the validity of the tool’s outputs, and a clear statement of which aspects of the study, article contents, data, or supporting files were affected/generated by AI tool usage (https://journals.plos.org/plosone/s/ethical-publishing-practice#loc-artificial-intelligence-tools-and-technologies)).*

**Response:** We expanded the dedicated AI disclosure. It now identifies qwen-plus through DashScope as the experimental model/provider and OpenAI Codex as the documented tool used for scenario wording, paraphrase generation, and preliminary annotation during dataset preparation; the exact historical Codex model version is not documented and was not guessed. It separately describes OpenAI ChatGPT/Codex assistance with code, audits, language, references, release preparation, and Word generation, the materials affected, author validation, and the fact that AI did not replace blinded expert adjudication, enter evaluator-only Ground Truth, alter frozen observations or statistics, authorize silent reruns, or qualify for authorship.

**Changes in the revised manuscript:** Methods, Section 3.16, paragraph beginning ‘Three distinct uses of AI are disclosed’ and the following paragraph.

**Journal Requirements — Point 3**

**Comment (verbatim):** *Please provide additional details regarding participant consent. In the ethics statement in the Methods and online submission information, please ensure that you have specified (1) whether consent was informed and (2) what type you obtained (for instance, written or verbal, and if verbal, how it was documented and witnessed). If your study included minors, state whether you obtained consent from parents or guardians. If the need for consent was waived by the ethics committee, please include this information. If you are reporting a retrospective study of medical records or archived samples, please ensure that you have discussed whether all data were fully anonymized before you accessed them and/or whether the IRB or ethics committee waived the requirement for informed consent. If patients provided informed written consent to have data from their medical records used in research, please include this information.*

**Response:** We clarified the ethics and consent scope without implying a pre-study review. During revision, the relevant institutional office at Beijing Information Technology College confirmed in writing that this professional expert-annotation activity was outside the scope of human-subject research requiring ethics review. No human research participants were enrolled as study subjects; the experts were annotators/adjudicators, and their personal, clinical, physiological, or behavioral characteristics were not analyzed as outcomes. Only deidentified expertise descriptors and aggregate agreement statistics are reported, with no reviewer names or identifiable comments in the manuscript or public archive. Participant informed-consent procedures were therefore not applicable. We do not claim a pre-study or retrospective approval, consent waiver, or approval/waiver number.

**Changes in the revised manuscript:** Methods, Section 3.17, paragraph beginning ‘During revision, the relevant institutional office’.

**Journal Requirements — Point 4**

**Comment (verbatim):** *Please note that PLOS One has specific guidelines on code sharing for submissions in which author-generated code underpins the findings in the manuscript. In these cases, we expect all author-generated code to be made available without restrictions upon publication of the work. Please review our guidelines at https://journals.plos.org/plosone/s/materials-and-software-sharing#loc-sharing-code and ensure that your code is shared in a way that follows best practice and facilitates reproducibility and reuse.*

**Response:** We verified that Zenodo DOI 10.5281/zenodo.22029432 is publicly published and updated the manuscript/online wording accordingly. The record provides the retained observations, manifests, author-generated scientific code, and analysis code needed to audit and reproduce the reported scientific decision and statistical analyses. Redistribution-restricted proprietary vendor assets and mappings remain explicitly excluded and are not required for those analyses.

**Changes in the revised manuscript:** Methods, Section 3.9, paragraph beginning ‘The provider returned the requested model identifier’; Discussion, Section 5.6.

**Journal Requirements — Point 5**

**Comment (verbatim):** *If the reviewer comments include a recommendation to cite specific previously published works, please review and evaluate these publications to determine whether they are relevant and should be cited. There is no requirement to cite these works unless the editor has indicated otherwise.*

**Response:** We evaluated both suggested papers and verified their bibliographic records and DOIs. We added them only to support the rapid development of adjacent intelligent-vehicle decision, prediction, planning, and control research, while explicitly preserving the distinction between those physical-decision layers and our semantic-assurance boundary.

**Changes in the revised manuscript:** Related Work, Section 2.1, paragraph beginning ‘Vehicle-focused language-model research now spans’; References 45–46.

REVIEWER #1

===========

**Reviewer #1 — Point 1**

**Comment (verbatim):** *The Safety Validator plays a central role in the proposed architecture, but its effectiveness strongly depends on the predefined Action Ontology and Safety Policy. The authors should explain more clearly how these rules were constructed and whether they can be systematically extended when new vehicle functions, diagnostic conditions, or semantic actions are introduced. This is important for understanding the scalability of the proposed safety-assurance mechanism.*

**Response:** We added the construction provenance and a prospective extension workflow. The ontology/policy are described as versioned research specifications derived from the task boundary, Pilot error analysis, frozen schema/aliases, and explicit required/forbidden rules. The documented role of the domain experts was Ground Truth annotation and adjudication; we therefore do not claim a separate expert validation of the ontology or policy. The prospective workflow covers schema/aliases, preconditions, required/forbidden combinations, conflict/precedence, fallback eligibility, domain-expert review of new additions, tests, versioning, refreezing, and independent revalidation. Unregistered semantics remain unknown and non-executable, and previously unseen functions were not validated here.

**Changes in the revised manuscript:** Methods, Section 3.2, new paragraph beginning ‘The Action Ontology and Safety Policy were research-constructed’; Discussion, Section 5.2.

**Reviewer #1 — Point 2**

**Comment (verbatim):** *The Risk Assessment module uses several fixed thresholds, such as battery SOC, driving duration, and driver-state confidence. The authors should provide more justification for these threshold values and discuss whether the resulting risk classification may change significantly under different vehicle types, driver populations, or operational environments.*

**Response:** We clarified that the thresholds are prespecified operational anchors for the frozen benchmark, not universal physiological, clinical, regulatory, or control limits; they were not optimized on Formal outcomes; and no sensitivity analysis was performed. The manuscript now states: ‘Their methodological role was to provide transparent deterministic stratification within the controlled benchmark; the study did not seek to validate the numerical cut points themselves.’ Transfer requires prospective calibration, sensitivity analysis, and a new frozen validation across the relevant vehicle, population, sensors, jurisdiction, and operational design domain.

**Changes in the revised manuscript:** Methods, Section 3.3, new paragraph beginning ‘These thresholds were prespecified operational anchors’; Discussion, Section 5.6.

**Reviewer #1 — Point 3**

**Comment (verbatim):** *The Runtime Fallback is intentionally limited to a predefined set of represented failure classes. However, practical multi-agent systems may experience compound faults, unexpected combinations of unavailable agents, conflicting states, or failures outside the predefined ontology. The authors should discuss more clearly how the framework behaves when an unrecognized or simultaneous failure occurs and what the safe default response would be in such situations.*

**Response:** We now state directly that unknown, simultaneous, compound, arbitrary, and out-of-ontology failures were not experimentally validated. We distinguish the present measured recovery from a future design requirement for fail-closed withholding, trace preservation, and escalation to a human or independently verified subsystem.

**Changes in the revised manuscript:** Methods, Section 3.6, new paragraph beginning ‘Unknown, simultaneous, compound, and arbitrary failures’; Discussion, Sections 5.3 and 5.6.

**Reviewer #1 — Point 4**

**Comment (verbatim):** *The proposed architecture is evaluated using one LLM/provider configuration, while the behavior of multi-agent reasoning can vary substantially across different models and future provider updates. The authors should discuss the extent to which the proposed routing, arbitration, Safety Validator, and Runtime Fallback mechanisms are model-independent and which parts may need to be redesigned when another LLM family is used.*

**Response:** We distinguished architectural portability from empirical generalizability. The dispatcher, schema, arbitration interface, validator, and fallback can be separated from a particular LLM, but response quality, schema compliance, disagreement, latency, and failure frequency may change. A replacement model/provider is therefore a new frozen configuration requiring repeated validation before any performance estimate is transferred.

**Changes in the revised manuscript:** Methods, Section 3.9, new paragraph beginning ‘The dispatcher, canonical schema, arbitration interfaces’; Discussion, Section 5.6.

**Reviewer #1 — Point 5**

**Comment (verbatim):** *The study defines safety mainly at the semantic-action level and explicitly separates this from trajectory or physical vehicle safety. This boundary is reasonable, but the manuscript should further clarify how semantic safety requirements would be transferred to downstream planning and control modules in a practical intelligent vehicle. In particular, it would be useful to discuss what additional assurance mechanisms would be required before a validated semantic action could influence physical vehicle behavior.*

**Response:** We added an explicit semantic-to-control assurance chain. The revised manuscript states: ‘A validated semantic action should be interpreted as an upstream contract, not as a direct execution authorization.’ Downstream use requires versioned mapping, planner constraints, control-level assurance, protocol/actuator acknowledgement, execution feedback, vehicle-state verification, fault monitoring, and lifecycle/configuration controls. None of these downstream assurances was established here.

**Changes in the revised manuscript:** Discussion, Section 5.5, paragraph beginning ‘A validated semantic action should be interpreted as an upstream contract’.

**Reviewer #1 — Point 6**

**Comment (verbatim):** *The HIL study mainly demonstrates preservation of the semantic-to-engineering boundary, while the available evidence for downstream execution is intentionally limited. The authors should provide a clearer discussion of what the HIL results actually establish regarding practical deployment and what remains outside the current validation scope. This would help distinguish semantic decision assurance from engineering execution reliability and avoid overly broad interpretation of the runtime-assurance concept.*

**Response:** We tightened the HIL interpretation. The revision states that the one-device, isolated-sandbox study establishes trace completion and preservation of a fail-closed semantic-to-engineering boundary only. Because no permitted mapped command occurred, permitted-command delivery reliability is not estimable; there was no protocol/actuator acknowledgement or physical vehicle outcome. We also identify the prospective evidence needed for broader engineering claims.

**Changes in the revised manuscript:** Results, Section 4.5; Discussion, Sections 5.5–5.6; Conclusions.

**Reviewer #1 — Point 7**

**Comment (verbatim):** *The background should highlight the rapid development of the intelligent vehicle, thereby enhancing the research value of this paper. Some recent work, such as “Enhancing High-Speed Cruising Performance of Autonomous Vehicles Through Integrated Deep Reinforcement Learning Framework, IEEE Transactions on Intelligent Transportation Systems, vol. 26, no. 1, pp. 835-848, Jan. 2025”, and “Interaction-Aware Trajectory Prediction for Safe Motion Planning in Autonomous Driving: A Transformer-Transfer Learning Approach, IEEE Transactions on Intelligent Transportation Systems, vol. 26, no. 10, pp. 17080-17095, Oct. 2025” can be referred.*

**Response:** We added both verified papers and used them narrowly to demonstrate rapid progress in integrated autonomous-vehicle decision/planning/control and interaction-aware trajectory prediction. The text explicitly notes that our semantic-assurance contribution operates upstream and does not inherit physical-control guarantees from those studies.

**Changes in the revised manuscript:** Related Work, Section 2.1; References 45–46.

REVIEWER #2

===========

**Reviewer #2 — Point 1**

**Comment (verbatim):** *However, the evidence is limited by an author-constructed synthetic benchmark and relatively weak comparators.*

**Response:** We agree and have made the benchmark/comparator limitation more prominent. The revised manuscript identifies the benchmark and both comparators as research-constructed controls within a common semantic interface, not exhaustive state-of-the-art production baselines, and explicitly states that the results do not establish general multi-agent superiority.

**Changes in the revised manuscript:** Methods, Section 3.4, paragraph beginning ‘The Single LLM comparator made one model call’; Discussion, Sections 5.1 and 5.6.

**Reviewer #2 — Point 2**

**Comment (verbatim):** *There is possible overlap between the Safety Validator and the Ground Truth policy, which may inflate the reported improvement.*

**Response:** We address this directly by distinguishing specification-level alignment from procedural and informational independence. The Safety Validator and Ground Truth intentionally share the frozen action/requirement/forbidden-action specification because policy compliance is the endpoint; that alignment defines the benchmark target and is not an independent external safety criterion. Experts independently annotated/adjudicated and froze the oracle before Formal execution, while the Validator received no Scenario ID, oracle field, draft annotation, method output, or evaluator query path. All three methods were evaluated against the same frozen evaluator oracle. Accordingly, the endpoint measures enforcement within the prespecified specification rather than external safety validity.

**Changes in the revised manuscript:** Methods, Sections 3.5 and 3.8; Discussion, Sections 5.2 and 5.6.

**Reviewer #2 — Point 3**

**Comment (verbatim):** *The HIL validation is limited by a small sample size, lack of permitted mapped HIL commands, and no real physical vehicle actuation.*

**Response:** We agree. The revised HIL text foregrounds the one-device/sandbox design, 30-case sample, zero applicable permitted mapped commands, non-estimable delivery endpoint, and absence of physical acknowledgement or actuation. HIL is interpreted as boundary-preservation evidence, not deployment validation.

**Changes in the revised manuscript:** Results, Section 4.5; Discussion, Sections 5.5–5.6; Conclusions.

**Reviewer #2 — Point 4**

**Comment (verbatim):** *Reproducibility is also limited by dependence on a non-versioned commercial LLM API.*

**Response:** We added the precise reproducibility limitation: the provider exposed no immutable qwen-plus snapshot, so the public archive supports audit and offline reproduction from retained responses but cannot guarantee bitwise regeneration through a future commercial-API call. Model/provider changes require a new frozen configuration and revalidation.

**Changes in the revised manuscript:** Methods, Section 3.9; Discussion, Section 5.6.

**Reviewer #2 — Point 5**

**Comment (verbatim):** *The results support bounded semantic policy enforcement under the tested scenarios only.*

**Response:** We tightened the Abstract, limitations, HIL discussion, and final Conclusion. The supported claim is now explicitly limited to enforcement of a frozen semantic policy and recovery for represented failure classes in the tested benchmark. The revision expressly excludes broader vehicle-system safety, general runtime assurance, physical execution reliability, public-road/deployment readiness, and functional-safety certification.

**Changes in the revised manuscript:** Abstract; Discussion, Sections 5.3, 5.5, and 5.6; Conclusions.

CLOSING STATEMENT

We believe the revision addresses all journal requirements and reviewer concerns while keeping the scientific claims bounded to the prespecified semantic-decision task, frozen benchmark, represented failure classes, and isolated HIL boundary. We appreciate the opportunity to improve the manuscript.

Sincerely,

Wei Dang and Lijun Gao
