# Supplementary material for: Safety-constrained and runtime-assured LLM multi-agent decision making for intelligent vehicle systems
Source: PLoS One. 2026 Oct 30;21(10):e0357928. doi: 10.1371/journal.pone.0357928 (PMC13722150; doi:10.1371/journal.pone.0357928)
Supplement: Peer review history

# Peer review: Decision Letter 0

**DOI:** 10.1371/journal.pone.0357928.r001

**Authors:** Jinhao Liang

13 Sep 2026

Dear Dr. Dang,

Thank you for submitting your manuscript to PLOS One. After careful consideration, we feel that it has merit but does not fully meet PLOS One’s publication criteria as it currently stands. Therefore, we invite you to submit a revised version of the manuscript that addresses the points raised during the review process.

Please submit your revised manuscript by Nov 12 2026 11:59PM. If you will need more time than this to complete your revisions, please reply to this message or contact the journal office at plosone@plos.org. When you're ready to submit your revision, log on to https://www.editorialmanager.com/pone/ and select the 'Submissions Needing Revision' folder to locate your manuscript file.

- A letter that responds to each point raised by the academic editor and reviewer(s). You should upload this letter as a separate file labeled 'Response to Reviewers'.
- A marked-up copy of your manuscript that highlights changes made to the original version. You should upload this as a separate file labeled 'Revised Manuscript with Track Changes'.
- An unmarked version of your revised paper without tracked changes. You should upload this as a separate file labeled 'Manuscript'.

If you would like to make changes to your financial disclosure, please include your updated statement in your cover letter. Guidelines for resubmitting your figure files are available below the reviewer comments at the end of this letter.

If applicable, we recommend that you deposit your laboratory protocols in protocols.io to enhance the reproducibility of your results. Protocols.io assigns your protocol its own identifier (DOI) so that it can be cited independently in the future. For instructions see: https://journals.plos.org/plosone/s/submission-guidelines#loc-laboratory-protocols. Additionally, PLOS One offers an option for publishing peer-reviewed Lab Protocol articles, which describe protocols hosted on protocols.io. Read more information on sharing protocols at https://plos.org/protocols?utm_medium=editorial-email&utm_source=authorletters&utm_campaign=protocols.

As the corresponding author, your ORCID iD is verified in the submission system and will appear in the published article. PLOS supports the use of ORCID, and we encourage all coauthors to register for an ORCID iD and use it as well. Please encourage your coauthors to verify their ORCID iD within the submission system before final acceptance, as unverified ORCID iDs will not appear in the published article. *Only*  the individual author can complete the verification step; PLOS staff *cannot*  verify ORCID iDs on behalf of authors.

We look forward to receiving your revised manuscript.

Kind regards,

Jinhao Liang

Academic Editor

PLOS One

Journal Requirements:

When submitting your revision, we need you to address these additional requirements.

1. Please ensure that your manuscript meets PLOS ONE's style requirements, including those for file naming. The PLOS ONE style templates can be found at

https://journals.plos.org/plosone/s/file?id=wjVg/PLOSOne_formatting_sample_main_body.pdf and

https://journals.plos.org/plosone/s/file?id=ba62/PLOSOne_formatting_sample_title_authors_affiliations.pdf

2. We note that you have declared the use of generative AI in the preparation of your manuscript. Please note that contributions by artificial intelligence (AI) tools and technologies to a study or to an article’s contents must be clearly reported in a dedicated section of the Methods, or in the Acknowledgements section for article types lacking a Methods section. This section should include the name(s) of any tools used, a description of how the authors used the tool(s) and evaluated the validity of the tool’s outputs, and a clear statement of which aspects of the study, article contents, data, or supporting files were affected/generated by AI tool usage (https://journals.plos.org/plosone/s/ethical-publishing-practice#loc-artificial-intelligence-tools-and-technologies).

3. Please provide additional details regarding participant consent. In the ethics statement in the Methods and online submission information, please ensure that you have specified (1) whether consent was informed and (2) what type you obtained (for instance, written or verbal, and if verbal, how it was documented and witnessed). If your study included minors, state whether you obtained consent from parents or guardians. If the need for consent was waived by the ethics committee, please include this information.

If you are reporting a retrospective study of medical records or archived samples, please ensure that you have discussed whether all data were fully anonymized before you accessed them and/or whether the IRB or ethics committee waived the requirement for informed consent. If patients provided informed written consent to have data from their medical records used in research, please include this information.

4. Please note that PLOS One has specific guidelines on code sharing for submissions in which author-generated code underpins the findings in the manuscript. In these cases, we expect all author-generated code to be made available without restrictions upon publication of the work. Please review our guidelines at https://journals.plos.org/plosone/s/materials-and-software-sharing#loc-sharing-code and ensure that your code is shared in a way that follows best practice and facilitates reproducibility and reuse.

5. If the reviewer comments include a recommendation to cite specific previously published works, please review and evaluate these publications to determine whether they are relevant and should be cited. There is no requirement to cite these works unless the editor has indicated otherwise.

Additional Editor Comments:

Please revise the manuscript point-by-point according to the reviewers' comments. The authors are free to decide whether to cite the references recommended by the reviewers.

[Note: HTML markup is below. Please do not edit.]

Reviewers' comments:

Reviewer's Responses to Questions

**Comments to the Author**

1. Is the manuscript technically sound, and do the data support the conclusions?

Reviewer #1: Yes

Reviewer #2: Yes

**********

2. Has the statistical analysis been performed appropriately and rigorously?

Reviewer #1: Yes

Reviewer #2: Yes

**********

3. Have the authors made all data underlying the findings in their manuscript fully available?

The PLOS Data policy

Reviewer #1: Yes

Reviewer #2: Yes

**********

4. Is the manuscript presented in an intelligible fashion and written in standard English?

Reviewer #1: Yes

Reviewer #2: Yes

**********

Reviewer #1: The manuscript proposes SCI-Proposed-v1.0, a multi-agent semantic decision architecture for intelligent vehicle systems that separates LLM-based reasoning from an independent Safety Validator and a deterministic Runtime Fallback mechanism. The study focuses on canonical semantic actions rather than direct vehicle control and evaluates the framework through a frozen benchmark, ablation analysis, decision-stability tests, and isolated hardware-in-the-loop validation. The topic is timely and relevant, and the separation between generative reasoning, semantic safety assurance, and bounded runtime recovery is conceptually meaningful. However, several aspects related to the scope of the safety claims, construction of the semantic policy, generalization of the decision architecture, runtime-fallback coverage, and practical deployment assumptions require further clarification.

1.The Safety Validator plays a central role in the proposed architecture, but its effectiveness strongly depends on the predefined Action Ontology and Safety Policy. The authors should explain more clearly how these rules were constructed and whether they can be systematically extended when new vehicle functions, diagnostic conditions, or semantic actions are introduced. This is important for understanding the scalability of the proposed safety-assurance mechanism.

2.The Risk Assessment module uses several fixed thresholds, such as battery SOC, driving duration, and driver-state confidence. The authors should provide more justification for these threshold values and discuss whether the resulting risk classification may change significantly under different vehicle types, driver populations, or operational environments.

3.The Runtime Fallback is intentionally limited to a predefined set of represented failure classes. However, practical multi-agent systems may experience compound faults, unexpected combinations of unavailable agents, conflicting states, or failures outside the predefined ontology. The authors should discuss more clearly how the framework behaves when an unrecognized or simultaneous failure occurs and what the safe default response would be in such situations.

4.The proposed architecture is evaluated using one LLM/provider configuration, while the behavior of multi-agent reasoning can vary substantially across different models and future provider updates. The authors should discuss the extent to which the proposed routing, arbitration, Safety Validator, and Runtime Fallback mechanisms are model-independent and which parts may need to be redesigned when another LLM family is used.

5.The study defines safety mainly at the semantic-action level and explicitly separates this from trajectory or physical vehicle safety. This boundary is reasonable, but the manuscript should further clarify how semantic safety requirements would be transferred to downstream planning and control modules in a practical intelligent vehicle. In particular, it would be useful to discuss what additional assurance mechanisms would be required before a validated semantic action could influence physical vehicle behavior.

6.The HIL study mainly demonstrates preservation of the semantic-to-engineering boundary, while the available evidence for downstream execution is intentionally limited. The authors should provide a clearer discussion of what the HIL results actually establish regarding practical deployment and what remains outside the current validation scope. This would help distinguish semantic decision assurance from engineering execution reliability and avoid overly broad interpretation of the runtime-assurance concept.

7. The background should highlight the rapid development of the intelligent vehicle, thereby enhancing the research value of this paper. Some recent work, such as “Enhancing High-Speed Cruising Performance of Autonomous Vehicles Through Integrated Deep Reinforcement Learning Framework, IEEE Transactions on Intelligent Transportation Systems, vol. 26, no. 1, pp. 835-848, Jan. 2025”, and “Interaction-Aware Trajectory Prediction for Safe Motion Planning in Autonomous Driving: A Transformer-Transfer Learning Approach, IEEE Transactions on Intelligent Transportation Systems, vol. 26, no. 10, pp. 17080-17095, Oct. 2025” can be referred.

Reviewer #2: The manuscript presents a carefully structured LLM multi-agent semantic decision architecture with independent safety validation and runtime fallback.

However, the evidence is limited by an author-constructed synthetic benchmark and relatively weak comparators.

There is possible overlap between the Safety Validator and the Ground Truth policy, which may inflate the reported improvement.

The HIL validation is limited by a small sample size, lack of permitted mapped HIL commands, and no real physical vehicle actuation.

Reproducibility is also limited by dependence on a non-versioned commercial LLM API.

The results support bounded semantic policy enforcement under the tested scenarios only.

They do not establish broader vehicle-system safety, general runtime assurance, real-world deployment readiness, or functional-safety certification.

**********

what does this mean?). If published, this will include your full peer review and any attached files.

If you choose “no”, your identity will remain anonymous but your review may still be made public.

**Do you want your identity to be public for this peer review?**  For information about this choice, including consent withdrawal, please see our Privacy Policy

Reviewer #1: No

Reviewer #2: No

**********

[NOTE: If reviewer comments were submitted as an attachment file, they will be attached to this email and accessible via the submission site. Please log into your account, locate the manuscript record, and check for the action link "View Attachments". If this link does not appear, there are no attachment files.]

To ensure your figures meet our technical requirements, please review our figure guidelines: https://journals.plos.org/plosone/s/figures

You may also use PLOS’s free figure tool, NAAS, to help you prepare publication quality figures: https://journals.plos.org/plosone/s/figures#loc-tools-for-figure-preparation.

NAAS will assess whether your figures meet our technical requirements by comparing each figure against our figure specifications.

# Author comment: Author response to Decision Letter 1

**DOI:** 10.1371/journal.pone.0357928.r002

10 Oct 2026

Thank you for the careful and constructive comments from the Academic Editor and reviewers. We have revised the manuscript point by point and uploaded a detailed Response to Reviewers as a separate file. The revision addresses all journal requirements, all seven comments from Reviewer #1, and all five concerns raised by Reviewer #2. No experiments were rerun, and no frozen scientific observations, Ground Truth labels, sample sizes, statistical results, tables, figures, or Supporting Information values were changed. Please see the uploaded Response to Reviewers document for the complete point-by-point responses and corresponding manuscript changes.

Attachment Submitted filename: PONE-D-26-42132_Response_to_Reviewers.docx

# Peer review: Decision Letter 1

**DOI:** 10.1371/journal.pone.0357928.r003

**Authors:** Jinhao Liang

15 Oct 2026

Safety-constrained and runtime-assured LLM multi-agent decision making for intelligent vehicle systems

PONE-D-26-42132R1

Dear Dr. Dang,

We’re pleased to inform you that your manuscript has been judged scientifically suitable for publication and will be formally accepted for publication once it meets all outstanding technical requirements.

Within one week, you’ll receive an e-mail detailing the required amendments. When these have been addressed, you’ll receive a formal acceptance letter and your manuscript will be scheduled for publication.

An invoice will be generated when your article is formally accepted. Please note, if your institution has a publishing partnership with PLOS and your article meets the relevant criteria, all or part of your publication costs will be covered. Please make sure your user information is up-to-date by logging into Editorial Manager at Editorial Manager® and clicking the ‘Update My Information' link at the top of the page. For questions related to billing, please contact billing support.

If your institution or institutions have a press office, please notify them about your upcoming paper to help maximize its impact. If they’ll be preparing press materials, please inform our press team as soon as possible -- no later than 48 hours after receiving the formal acceptance. Your manuscript will remain under strict press embargo until 2 pm Eastern Time on the date of publication. For more information, please contact onepress@plos.org.

Kind regards,

Jinhao Liang

Academic Editor

PLOS One

Additional Editor Comments (optional):

Reviewers' comments:

Reviewer's Responses to Questions

**Comments to the Author**

Reviewer #1: All comments have been addressed

Reviewer #2: All comments have been addressed

**********

2. Is the manuscript technically sound, and do the data support the conclusions?

Reviewer #1: Yes

Reviewer #2: Yes

**********

3. Has the statistical analysis been performed appropriately and rigorously?

Reviewer #1: Yes

Reviewer #2: N/A

**********

4. Have the authors made all data underlying the findings in their manuscript fully available?

The PLOS Data policy

Reviewer #1: Yes

Reviewer #2: Yes

**********

5. Is the manuscript presented in an intelligible fashion and written in standard English?

Reviewer #1: Yes

Reviewer #2: Yes

**********

Reviewer #1: (No Response)

Reviewer #2: Proofreading of the manuscript required. This will help improve the english by removing any grammatical mistakes and spelling mistakes if any.

The references need to be cross check as well

**********

what does this mean?). If published, this will include your full peer review and any attached files.

If you choose “no”, your identity will remain anonymous but your review may still be made public.

**Do you want your identity to be public for this peer review?**  For information about this choice, including consent withdrawal, please see our Privacy Policy

Reviewer #1: No

Reviewer #2: No

**********

# Editor report: Acceptance letter

**DOI:** 10.1371/journal.pone.0357928.r004

**Authors:** Jinhao Liang

PONE-D-26-42132R1

PLOS One

Dear Dr. Dang,

I'm pleased to inform you that your manuscript has been deemed suitable for publication in PLOS One. Congratulations! Your manuscript is now being handed over to our production team.

At this stage, our production department will prepare your paper for publication. This includes ensuring the following:

* All references, tables, and figures are properly cited

* All relevant supporting information is included in the manuscript submission,

* There are no issues that prevent the paper from being properly typeset

You will receive further instructions from the production team, including instructions on how to review your proof when it is ready. Please keep in mind that we are working through a large volume of accepted articles, so please give us a few days to review your paper and let you know the next and final steps.

Lastly, if your institution or institutions have a press office, please let them know about your upcoming paper now to help maximize its impact. If they'll be preparing press materials, please inform our press team within the next 48 hours. Your manuscript will remain under strict press embargo until 2 pm Eastern Time on the date of publication. For more information, please contact onepress@plos.org.

You will receive an invoice from PLOS for your publication fee after your manuscript has reached the completed accept phase. If you receive an email requesting payment before acceptance or for any other service, this may be a phishing scheme. Learn how to identify phishing emails and protect your accounts at https://explore.plos.org/phishing.

If we can help with anything else, please email us at customercare@plos.org.

Thank you for submitting your work to PLOS One and supporting open access.

Kind regards,

PLOS One Editorial Office Staff

on behalf of

Dr. Jinhao Liang

Academic Editor

PLOS One
